# Lactic Acid Bacteria Exopolysaccharides Producers: A Sustainable Tool for Functional Foods

**DOI:** 10.3390/foods10071653

**Published:** 2021-07-17

**Authors:** Roberta Prete, Mohammad Khairul Alam, Giorgia Perpetuini, Carlo Perla, Paola Pittia, Aldo Corsetti

**Affiliations:** 1 Faculty of Bioscience and Technology for Food, Agriculture and Environment, University of Teramo, 64100 Teramo, Italy; rprete@unite.it (R.P.); mkalam@unite.it (M.K.A.); ppittia@unite.it (P.P.); acorsetti@unite.it (A.C.); 2 Dalton Biotecnologie srl, Spoltore, 65010 Pescara, Italy; laboratorio@dalton.it

**Keywords:** lactic acid bacteria, exopolysaccharides, functional foods, dairy industrial applications, health benefits

## Abstract

Lactic acid bacteria (LAB) used in the food industry, mainly for the production of dairy products, are able to synthetize exopolysaccharides (EPS). EPS play a central role in the assessment of rheological and sensory characteristics of dairy products since they positively influence texture and organoleptic properties. Besides these, EPS have gained relevant interest for pharmacological and nutraceutical applications due to their biocompatibility, non-toxicity and biodegradability. These bioactive compounds may act as antioxidant, cholesterol-lowering, antimicrobial and prebiotic agents. This review provides an overview of exopolysaccharide-producing LAB, with an insight on the factors affecting EPS production, their dairy industrial applications and health benefits.

## 1. Introduction

Bacteria are well known for their ability to produce a wide variety of polysaccharides, which can be tightly linked to the cell surface forming a capsular polysaccharide (CPS) or secreted as exopolysaccharides (EPS). Exopolysaccharides have a high molecular weight and biodegradable polymers formed by monosaccharide residues of sugar and sugar derivatives, and are produced by a wide range of bacteria [1]. Different bacterial groups—mainly lactic acid bacteria (LAB) and bifidobacterial—produce a wide variety of carbohydrate polymers during fermentation. Although the beneficial effect that EPS production provides to the bacterial physiology is still not entirely clarified, it seems that LAB produce EPS as a protective matrix to endure all the stresses related to fermentation processes, such as pH, temperature and osmotic stress, among others [2]. Moreover, EPS play a fundamental role in formation of biofilm matrix, and cell aggregation or adhesion mechanisms to both the abiotic and biotic surface (i.e., intestinal mucosa). EPS deeply regulate microbial life, being involved in several ecological process that enhance bacterial colonization of either technological or gastrointestinal microenvironment, through a stable cell recognition and cooperation and by acting as a protective barrier against harmful substances (i.e., antibiotics, toxic compounds), that ultimately lead to increased bacterial survival [3].

LAB produce a wide variety of EPS with different chemical structure and physical characteristics that, in turn, affect their technological properties that result in their main interest for food industry applications. For instance, the side chains found on many linear polysaccharides promote conformational disorder, resulting in solubility in aqueous solutions. Thus, xanthan, which possesses a cellulose backbone with trisaccharide side chains on alternate glucose residues, has been described as a natural water-soluble cellulose derivative. The EPS XM6 produced by *Enterobacter* spp. is made up of a tetrasaccharide-repeating unit and lacks acylation. It interacts with various monovalent and divalent cations to form a gel. However, the EPS 54 released by *K. aerogenes* has the same structure but failed to gel [4]. 

The precise EPS physiological function is still not fully understood in terms of different EPS types and producer strains. They play a central role in affecting the rheological and sensory characteristics of dairy products since they increase viscosity and related organoleptic properties at consumption. In the cereal and bakery industry they can replace food gums such as hydroxypropylmethylcellulose (HPMC) [5]. Moreover, they are involved in bacterial–host interactions and microbial-mediated immunomodulation [6]. EPS help producing bacteria to face gastrointestinal stresses and to persist longer in the gut [7]. These health benefits allow the production of added-value, functional products in line with consumers’ demand for healthy and “green” products [8]. The roles of some HoPS and HePS are reported in Table 1.

**Figure 1:**
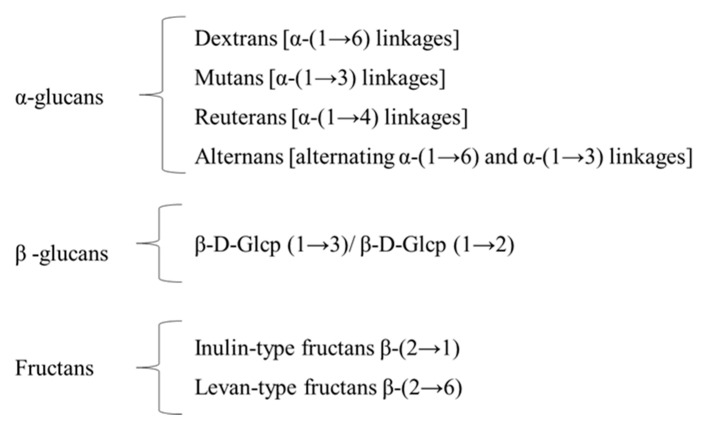
Classification of HoPS. The majority of HoPS-producing LAB produce a single glycansucrase enzyme, but some contain more than one glycansucrase gene and therefore may synthesize more than one type of HoPS.

EPS can be divided based of repeating units’ composition into two groups: (i) homopolysaccharides (HoPS) and (ii) heteropolysaccharides (HePS). The former (HoPs) present only one type of monosaccharide (D-glucose or D-fructose), and the main ones are α-glucans, β-glucans or fructans. They can be further classified on the basis of the glycosyl type, linkage variety and the position of carbon involved in the bond (Figure 1). They are produced by different genera of LAB, including *Weissella*, *Leuconostoc*, *Lactobacillus* (recently reclassified into 25 genera) [9], *Pediococcus* and *Streptococcus*.

HePS contain from three to eight repeating units made up of two or more monosaccharides (e.g., rhamnose, fructose, galactose or glucose); they can present some molecular modifications, including acetylations, pyruvylations and phosphorylations [10]. Van Kranenburg et al. [11] and Mozzi et al. [12] described the production of HePS containing a pentameric repeating unit of galactose in a *Lactococcus (Lact.) lactis* subsp. *lactis* H414 and *Lactobacillus (Lb.) delbrueckii* subsp. *bulgaricus* CRL 406 and 142. 

## 2. Bacterial Synthesis of EPS

### 2.1. HoPS Biosynthetic Pathways

HoPS are produced in the extracellular environment from a molecule of sucrose, which is used as donor of a monosaccharide by action of an extracellular enzyme belonging to the glycosyl hydrolase family [13]. α-Glucans and β-fructans are then produced through glucansucrases and fructansucrase, respectively. These enzymes hydrolyze the sucrose–glycosidic bond and use the energy released to transfer the glucosyl or the fructosyl moieties to the growing reducing end of the polymer [13,14]. α-Glucans are generally produced by several LAB genera, and the main are *Leuconostoc*, *Lactobacillus*, *Lactiplantibacillus*, *Limosilactobacillus*, *Lacticaseibacillus*, *Streptococcus*, *Pediococcus* and *Weissella*. 

β-Glucans production occurs in the intracellular environment by a membrane-associated glucosyltransferase, and it does not use sucrose as substrate. Levan-type and inulin-type fructans are produced by levansucrases and inulosucrases, respectively. β-Glucans production has been described in LAB isolated from alcoholic beverages, such as *Pediococcus* spp., *Lactiplantibacillus* spp. and *Oenococcus* spp. Fructans are produced by *W. confusa* strains, *Limosilactobacillus (Lim.) reuteri* Lb 121 and a *Lim. pontis* strain. Inulin production has been reported only in few strains of *Lactiplantibacillus*, *Leuconostoc* and streptococci [13]. Leemhuis et al. [15] described the presence of a cell-associated transglycosylating (GH70) 4,6-α-glucanotransferase in a strain of *Lim. reuteri.* This enzyme catalyzes the synthesis of α-glucan from starch and maltodextrins. 

### 2.2. HePS Biosynthetic Pathways

**Figure 2:**
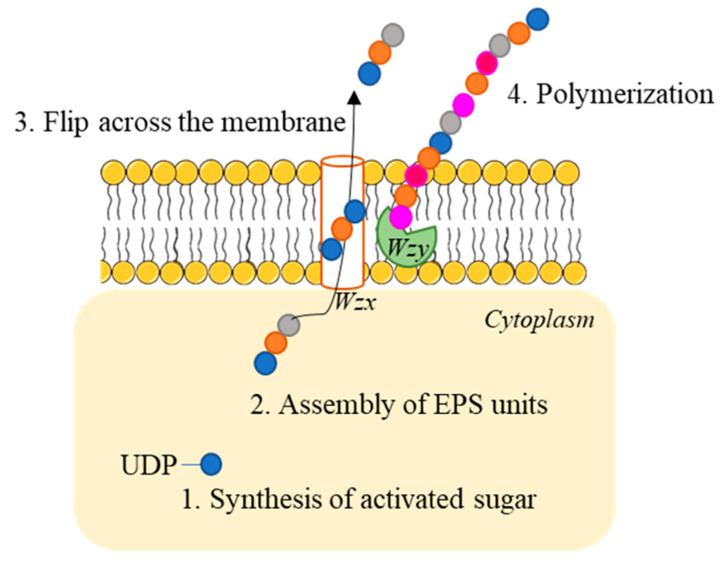
HePS production via Wzy-dependent pathway.

HePS production is more complex than HoPS one. Repeating units are synthetized intracellularly and polymerized extracellularly (Figure 2). 

The main pathway used by LAB to produce HePS is the Wzy-dependent pathway. This process can be divided into 4 steps:

(1) Activated sugar generation: Sugar nucleotides are produced from the glucose-6-phosphate, fructose 6-phosphate or glucose-1-phosphate produced in the Leloir pathway during lactose catabolism [15]. This reaction is catalyzed by priming-GT (a membrane bound polyprenyl-P sugar-1-P transferase).
(2) Assembly of EPS units: sugar nucleotides are added via membrane-bound GT.
(3) Transport across the membrane: repeating units are flipped across the membrane via a flippase (Wzx). This enzyme is bound to the membrane and shows 12 transmembrane domains.
(4) Polimerization: it is catalysed by the Wzy polymerase, which adds single repeating units generating a new glycosidic bond to the reducing end of the chain.

This pathway require energy and therefore is subjected to a strict regulation. For instance, in *S. thermophilus* and *Lb. bulgaricus* HePS production is favored under optimal growth conditions; in *Lacticaseibacillus (Lcb.) casei*, medium composition influenced exocellular polysaccharide production and its sugar composition. Polysaccharide production is also influenced by pH, temperature, aminoacids, vitamins and minerals [16].

### 2.3. Genetic of EPS Production

Genes of the Wzy-dependent pathway have been reported only in *Weisella*, *Leuconostoc*, *Lactiplantibacillus*, *Pediococcus* and *Streptococcus* genera. These genes are organized in an operon, the so-called *eps* operon, and can reside on a plasmid or the chromosome. Genes in the *eps* operon are grouped on the basis of their established or putative roles: modulatory genes (e.g., phosphoregulatory module *epsBCD*), polysaccharide assembly machinery genes (initiation *epsE*, polymerization *wzy*, flippase *wzx* and attachment *epsA*), genes encoding the GT necessary for the assembly of the repeating units and genes involved in the synthesis of activated sugar precursors and modification of the sugar residues [16]. 

In LAB, a typical *eps* gene cluster presents 5 highly conserved genes named *epsA*, *epsB*, *epsC*, *epsD* and *epsE*, and a more variable region including the polymerase *wzy*, the flippase *wzx*, and glucosyltransferases. In *Lpb. plantarum,* multiple EPS clusters have been described [17]. However, in lactobacilli, the EPS gene cluster architecture is more complex than that observed in other species such as *Lactococcus lactis* and *S. thermophilus*. 

For instance, the sequenced strain WCFS1 harbours four chromosomal clusters of *eps* genes. Two of them (*cps*2A-J and *cps*4A-J) are involved in capsular polysaccharide formation, while *cps*1A-I and *cps*3A-J seem to have a regulatory function and encode a priming glycosyl-transferase [18]. The most conserved cluster appears to be the *cps*4A-J, which presents tyrosine kinases, phosphotyrosine phosphatase, a priming glycosyltransferase, glycosyltransferases, a flipase and a polysaccharide polymerase [17].

In *Lact. lactis* the *eps* genes are named *epsR*, *epsA*, *epsB*, *epsC* and *epsD*, in *S. thermophilus, epsA*, *epsB*, *epsC*, *epsD* and *epsE*, while in *Lb. bulgaricus, epsABCDE* [19,20]. Several LAB species also contain a second cluster for the production of cell wall polysaccharides (CW-PS) anchored to peptidoglycan. These molecules contain rhamnose and have multiple functions. In *S. thermophilus*, they are involved in antibiotic stress response, while in *Lact. Lactis*, they act as phage receptors [21,22,23]. CW-PS production is regulated by *rmld*-associated gene clusters which contains from 12 to 27 genes with putative functions including GT, polysaccharide biosynthesis proteins, rhamnose biosynthesis proteins (RmlABCD) and transport molecules [21].

## 3. EPS-Producing Lactic Acid Bacteria

The ability to produce EPS by LAB depends on several factors, and the amount of production is largely species- and strain-specific (Table 2). 

In general, in non-optimized conditions it has been reported that EPS yield is under 1 g/L for the majority of LAB strains [24], such as *Lim. fermentum* (0.75–0.85 g/L) [25,26], *Lev. brevis* (0.35 g/L) [27], *Lpb. plantarum* (0.14–0.4 g/L) [27,28] and *Lact. lactis* (0.2–0.35 g/L). However, a higher amount of EPS has been reported for *Lcb. rhamnosus* RW-9595M (2.8 g/L) [29], the *Lb. kefiranofaciens* WT-2B strain (2.5 g/L) [30], the *Lpb. plantarum* BR2 strain (2.8 g/L) [31] and *Lim. reuteri* L26 *and Lim. reuteri* DSM 17938 (4.3–5 g/L) [32]. 

Physicochemical parameters influencing bacterial growth (i.e., temperature, incubation time, pH, oxygen rate, etc.) as well as different carbon and nitrogen sources are the main factors affecting EPS production (Table 3) [8,53]. Moreover, the same LAB strain can synthetize various EPS under different growth conditions [54]. The composition of the culture medium, including added nutrients as growth enhancers, is one of the most important factors affecting EPS production [55]. For instance, de Man Rogosa and Sharpe (MRS) media supplemented with sucrose, fructose or maltose is generally used to screen HoPS production [37,55,56].

Several other authors also reported the impact of culture medium composition on the level of EPS produced by LAB [34,37,42,55,57]. In this regard, a number of media specifically developed for EPS production, including skim or partially skim milk, whey based medium, semi-defined medium, chemically defined medium and basal minimum medium have also been applied [58]. Among them, milk-based media have shown to be good substrates to be used for EPS production, especially after enrichment with an appropriate carbon source. *Lb. delbrueckii* subsp. *bulgaricus* showed a high amount of EPS production ca. (5.5 g/L) in modified skim milk with glucose as a carbon source [42]. Additionally, *S. thermophilus* has been found to exhibit high EPS production in milk medium in comparison to MRS or M17 broths [59]. On the other hand, *Lcb. casei* and *Lb. delbreuckii* subsp. *bulgaricus* strains cultured in fermented milk produced a lower amount of EPS (<600 mg/L) compared to M17 broth supplemented with different carbon and nitrogen sources (1500 mg/L) [60]. Organic nitrogen (i.e., casein, tryptone, yeast extract, peptone) significantly improved the production of specific EPS, such as kefiran, while the use of inorganic nitrogen, such as ammonium chloride, leads to a lower amount of kefiran grains [61,62]. Physicochemical parameters are also relevant for LAB in growing and producing EPS. The optimal pH value for production of EPS is generally considered to be around pH 6.0 but varies greatly between species and LAB strains [63]. The *S. thermophilus* ST111 strain was found to produce EPS at maximum capacity when the pH of the culture medium was maintained at 6.2 [57]. For *Lb. delbrüeckii* subsp. *bulgaricus*, the ideal pH value was considered to be 6.5 [54], as found also for the *Lim. fermentum* F6 strain [64]. Some authors reported that low pH (i.e., 4.9) significantly affects EPS degradation, leading to a decrease in EPS yields [65]. 

Regarding temperature, the hydrolytic degradation of EPS can be reduced using sub-optimal temperature of incubation (18–25 °C for mesophilic and 35–37 °C for thermophilic species) [63,66]. In turn, several studies reported that sub-optimal temperature is the optimal temperature for higher EPS production [59,67], probably due to the physiological stress induced by the reduced temperature on bacterial cells, particularly in strains lacking proteolytic activity (e.g., *S. thermophilus*) [57,59,68]. *S. thermophilus* BN1 showed an EPS production in skim milk 5-fold higher at 37 °C compared to that of 42 °C [59]. Likewise, *Lim. fermentum* F6, *Lim. fermentum* TDS030603 and *Lcb. casei* CRL 870 produced elevated amounts of EPS when incubated at 37 °C [64,69]. For the *Lb. delbrüeckii* subsp. *bulgaricus* DSM 20081 strain, the EPS yield was higher when incubated between 30 °C to 40 °C, whereas the EPS production was decreased by almost half when incubated at 45 °C [67]. On the other hand, a study by Ruas-Madiedo et al. [70] reported no effect of temperature on the EPS production when the *Lact. lactis* subsp. *cremoris* strain was used to ferment milk. 

The activity of EPS-producing bacteria can be also influenced by the concomitant presence of different LAB strains in the culture medium. This topic is of high significance since, in industrial practice, mixed cultures consisting of multiple strains of the same LAB species and/or different species are typically used. The synergic actions of *Lb. delbrüeckii* subsp. *bulgaricus* and *S. thermophilus* are well-known in yogurt-making. On the other hand, competition and/or growth inhibition may also occur. When a mixed culture was prepared containing the EPS-producer strain of *Lb. kefiranofaciens* ZW3 and non-EPS-producer strains of *Lb. delbrüeckii* subsp. *bulgaricus* and *S. thermophilus*, *Lb. kefiranofaciens* ZW3 was found to exhibit structurally different EPS compared to the structure of EPS in the absence of yogurt culture bacteria [71]. Therefore, the mixed culture of EPS-producer and non-producer strains can offer the opportunity to obtain EPS of a different type and structure and that, in turn, can lead to different qualitative properties in the final product. Recently, a study by Berstch et al. [72] investigated the effects of co-culture on the amount of EPS produced by three different *Lcb. rhamnosus* strains in combination with *Saccharomyces cerevisiae*, showing positive interactions for promoting EPS biosynthesis [72].

As stated above, different bacterial species/strains influence EPS production. Yu et al. [73] revealed that only a strain of *Weissella cibaria* isolated from kimchi produced up to 9.8 g/L of EPS in a dose-dependent way in response to a high sucrose supplementation in the growing media. Similarly, Zannini et al. [74] demonstrated that different strains of *W. cibaria* showed about 2.8-fold variance in EPS production in sucrose-MRS broth. The different behavior of strains in terms of EPS production has been corroborated by other studies. Different strains of *Lpb. plantarum* isolated from different sources—Turkish sourdough and cow milk—showed an average EPS production of 1153.8 µg/107 cells and 197 mg/L, respectively [75,76].

Abdalrahim et al. [77] showed that different strains of *L. pseudomesenteroides* and *L. mesenteroides* produced EPS with concentrations ranging from 18.08 to 61.9 g/L. *Bifidobacterium animalis, B. longum* and *B. pseudocatenulatum* isolated from human intestinal microbiota produced HePS made up of galactose and glucose. Some of the EPS produced also contained rhamnose in higher proportion compared to those produced by LAB isolated from food [78].

This different behaviour of LAB in terms of EPS production could be explained through changes in gene expression in response to stressing conditions. For instance, a glycosyltransferase related to EPS synthesis was upregulated after exposure to acidic stress, bile salts and osmotic stress in *B. animalis* subsp. *lactis* [79]. Other studies demonstrated this overexpression of genes related to EPS production in other species such as *S. thermophilus* [80] and *Lcb. paracasei* [81] when stressing conditions are encountered. 

### 3.1. In Vitro Screening of EPS-Producing LAB

Investigations on EPS-producing bacteria require an early characterization based on in vitro screening of LAB strains. There are a wide variety of microbiological techniques that can be utilized to screen EPS-producing strains as “ropy or non-ropy”. The ropy phenotype is distinguished visually by the formation of long filaments when a needle is lifted from the colony surfaces as well as from the cell pellet in fermented liquid [58]. Visual observation of viscosity in liquid media has also been used as a screening method for EPS producer strains [55]. However, not all viscous cultures exhibit a “ropy” phenotype. Ortega-Morales et al. [86] successfully screened EPS producer strains based on the “mucoid” and “slimy” appearance of strains. The terminology to describe the EPS-producing strains of LAB is confusing, and terms such as, “mucoid”, “slimy” and “ropy” have been often utilized imprecisely in literature [58]. In fact, the visual in vitro screening can lead to the selection of false negative strains and to mislead in defining the morphological characteristics of “ropy” colonies. Novel polymers probably will not be recognized because of a missing evident slime development. Furthermore, the presence of different carbohydrates as a carbon source can significantly affect the formation of ropy filaments, leading to misinterpretation of ropy phenotypes [55].

EPS producers can be identified applying other more objective tests. A common assay is ruthenium red stain in milk agar plates. Non-ropy colonies appear red, while ropy colonies from EPS producers remain white [87]. Aniline Blue fluorochrome (Sinofluor) interacts with β-(1-3)-glucans and has been used to screen 89 putative EPS producer strains [88]. Similarly, Lauer Cruz and de Souza da Motta [89] have successfully used a fluorescence-based method. Calcofluor White binds to both succinoglycan and pure β-(1-3)- and β-(1-4)-glucans and, when irradiated by long-wave UV radiation, exhibits a blue-green fluorescence. Fluorescence-negative colonies on agar-plates can thus easily be detected, especially if applied for the characterization of a large number of strains. However, the use of these dyes may not be suitable for the screening of novel polymers containing various sugars or uronic acids as well as deoxy-and amino-sugars, as interactions of the dyes with new EPS are uncertain, making this technique impractical for identifying novel variants of EPS.

Recently, with the development of omics technologies, the in vitro screening of EPS producers can benefit from the wide amount of complete genome data available for a variety of species to gain knowledge of genetic determinants and metabolic pathways behind the bacterial capacity to produce EPS [90]. The wide number of complete genome sequences for different species is useful to understand microbial biological capabilities and, for instance, can be exploited to optimize the EPS production in food industry bacteria. Whole-genome analysis of EPS clusters have been already reported for some species, such as *S. thermophilus* [91], *Lpb. plantarum* [92] and *Lact. lactis*; additionally, comparative genomic analyses for EPS biosynthesis have been performed for *Lb. bulgaricus* 2038 [93]. More information on the genome of the EPS producer strains will enable to develop additional strategies to improve EPS production, and to engineer strains in order to modify composition and chain length. Omic approaches are needed to control and optimize production in order to improve EPS yields. For instance, metagenomic studies have been applied to investigate the metabolic potential of LAB in the fermentation of kimchi [94] and the transcriptomic approach has been used for yogurt and milk fermentation [20,95]. Moreover, models based on the genome sequence could integrate transcriptome, metabolomics and proteomics data to predict EPS production at different conditions.

### 3.2. EPS Isolation and Purification

EPS isolation and purification are of paramount importance for the recovery and characterization of single EPS. Recently, the isolation methods were extensively reviewed for providing a more comprehensive outlook of recent developments on this subject [8,53]. 

Various techniques of isolation and purification have been developed. Generally, EPS isolation consists of the following required steps: (i) a centrifugation step to remove bacterial cells; (ii) EPS precipitation; (iii) EPS purification by dialysis against water; (iv) freeze-drying. 

In complex media with a high protein content (e.g., skim milk), and at acidic pH, purification and quantification of EPS requires the isolation of EPS from a protein network where EPS and bacterial cells are trapped or conjugated with the protein [53,96]. For instance, a heating step (e.g., at 100 °C for 15 or 30 min) as a pre-treatment prior to isolation can be applied to inactivate endogenous enzymes that can cause EPS degradation in the medium [53]. Then, proteins can be precipitated with trichloroacetic acid (TCA) [27,49,58,70] or a combination of TCA and proteases [97]. In certain cases, heat treatment is used to improve the EPS recovery from the culture medium as the first step in EPS isolation [27,32,35,58,97,98]. For the following precipitation of EPS, ethanol is commonly used [27,42,43,50,55,58,99], although isopropanol [100], acetone [33] or a mixture of acetone and ethanol [101] have been also used.

In general, after precipitation and recovery, the isolated EPS-rich phase is then dissolved in deionized water, and this dispersion is used for the removal of the protein fraction with tricholoracetic acid (TCA). To eliminate low-molecular-mass contaminating carbohydrates, the dissolved EPS is then dialyzed against water and a dialysis membrane with a cutoff of 12–14-kDa is commonly used [8]. As a preceding step to EPS purification, membrane filtration techniques such as microfiltration, ultrafiltration and/or diafiltration have also been used [50,58,70,101].

Finally, the EPS is lyophilized [27,33,35,43,99,101] to remove moisture and allow long storage. For molecular characterization purposes, additional purification steps have been employed to increase the purity of the EPS fraction, by applying advanced instrumental techniques such as size-exclusion chromatography (SEC) or ion-exchange chromatography [8,27,36,44,47,53,99].

### 3.3. EPS Quantification and Characterization

Gravimetric method, such as weighing the dry mass of the purified polysaccharide, is the most straightforward approach for quantifying EPS yield [58]. However, this is not very reliable, particularly when the isolated EPS fraction does contain low quantities of EPS and/or in presence of impurities [8]. 

A colorimetric procedure for the determination of total carbohydrate (expressed as glucose equivalents per unit of weight) by phenol-sulfuric acid method is the most widely used method of calculating EPS yield [27,33,34,42,43,44,49,55,99,102]. A similar colorimetric technique is the anthrone-sulfuric acid method reported by some researchers as another useful method for the quantification of carbohydrates [101,103,104]. However, these colorimetric methods can suffer interference with the other carbohydrates present in the growth medium [8,53].

Moreover, Ruijssenaars et al. [105] estimated more precisely the quantity of EPS by using the following formula:

EPS = TS − RS

where TS is the total sugar calculated by the phenol-sulfuric method and RS is the reducing sugar fraction determined by the dinitrosalicylic acid method. Traditionally, colorimetric assays of quantification represent the cheapest and simplest methods to be used but they are not free from interference that needs to be considered when quantitative information is needed. 

Instrumental methods have been developed to improve the quantification of EPS production. Tang et al. [44] used ion-exchange chromatography to separate EPS, and then the carbohydrate content of the EPS-rich fraction was estimated using the phenol-sulfuric method.

Size-exclusion chromatography can be applied also to more precisely determine the EPS concentration, and the quantification of EPS can be done through the corresponding elution peak using the refractive index measurements [27,53,58].

For a rapid and simultaneous quantification of lactic acid, lactose and EPS directly in culture media without a prior isolation step, a near infrared (NIR) spectroscopy method has also been reported by Macedo et al. [29] This method led to results with strong correlation (R^2^ > 0.90) with reference methods to indicate the effectiveness for monitoring the production of EPS and lactic acid during fermentation.

High-performance, anion-exchange chromatography coupled with pulsed amperometric detection [8,27,35,53] or gas chromatography (GC) [32,43] are commonly utilized to analyze the monosaccharide composition of EPS. To degrade the EPS to its constituent monosaccharides, which are then detected, an initial hydrolysis step is necessary. Trifluoroacetic acid (TFA), HCl or H_2_SO4 at high temperature (i.e., 100–121 °C) can be used for the hydrolysis of EPS. TFA is the most frequently used acid, also because the unreacted TFA can be easily eliminated by evaporation [58]. The resulting monosaccharides are then derivatized to alditol acetates and can be determined by HPLC or GC [8,27,32,35,43,53]. 

Enzyme hydrolysis as an alternative to acid hydrolysis has also been employed for the cleavage of specific linkage present in the EPS polymer [56,106]. The subsequent release of monosaccharide components indicates the existence of an enzyme-targeted linkage in the EPS polymer (e.g., dextranase derived hydrolysis of a polymer indicates the presence of α-1,6 linkages, and thus, the EPS could be a dextran) [56]. However, one drawback associated with this type of assay is the limited availability of suitable enzymes that can specifically act on a specific linkage predicted to be present in the EPS polymer [8].

Recently, anion-exchange chromatography and carbohydrate gel electrophoresis have been used to evaluate the presence of a charge on the EPS [49], while Fourier transform-infrared spectroscopy has been used to obtain information about functional groups in EPS, including the presence of charged moieties such as phosphate and sulfate groups [38,42,49].

Nuclear magnetic resonance (NMR) spectroscopy is the only analytical instrument that can allow to obtain information about the molecular arrangement of carbohydrates and to identify the structural characteristics of the polysaccharides [58]. NMR enables the interaction of carbon and hydrogen atoms with neighboring atoms, and chemical groups to be elucidated, allowing their relative location in the structure to be determined and thus the polymer configuration to be known [27,38,42,45].

Microscopic techniques can be used to detect EPS presence at the microstructural level in food matrices. In particular, after staining with a fluorescent lectin, confocal laser scanning microscopy analysis allowed to obtain both qualitative [99] and quantitative [79] information about EPS structure and production in fermented soy milk and fermented milks, respectively. Moreover, typically after staining with ruthenium red, scanning electron microscopy [107] or transmission electron microscopy [98] can be also used to visualize and confirm EPS production in situ. More in particular, in dairy fermentation studies, these microscopic staining techniques are the most commonly used as they confirm the in situ production without damaging the food microstructure and enable to evidence the interaction of EPS with other food components [8].

## 4. EPS and Health Benefits

Besides the application in food industry, thanks to the thickening and structuring abilities that allow an improvement of the texture and rheological properties of the products in which EPS are produced, LAB-derived EPS have also gained growing interest in other sectors (e.g., pharma, nutraceutical) due to their biocompatibility, non-toxicity and biodegradability features [108]. The EPS-producing bacteria, mainly LAB and bifidobacteria, known to be a reservoir of various probiotic strains, have long been used in the food industry thanks to their safety, as they are generally recognized as safe (GRAS) by the Food and Drug Administration (FDA) and are included in the quality presumption of safety (QPS) list of the European Food Safety Authority (EFSA) [6], and in recent years their use has been increasing as “natural” food additives with beneficial health effects.

**Figure 3:**
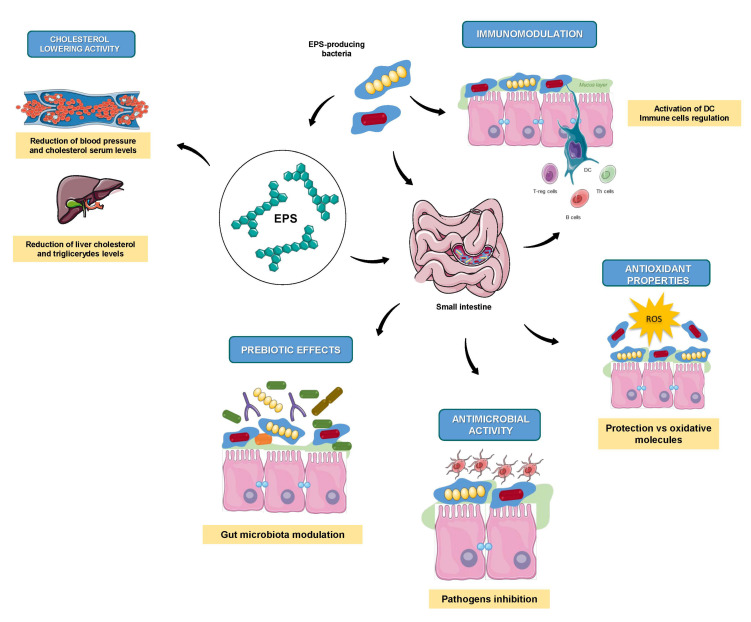
Principal health benefits of EPS produced by LAB strains. Graphical illustrations were created by using some graphical items from Servier Medical Art by Servier, available on https://smart.servier.com/ (accessed on 15 January 2021) under a Creative Commons Attribution 3.0 Unported License.

The health functionality of EPS is correlated with the specific molecular and structural properties (e.g., molecular weight, alignment and monosaccharide composition) that are affecting their bioactivity [109]. Moreover, it is worth noting that the specific chemical structure of EPS depends on both the bacteria involved and the conditions applied during the fermentation process, leading to the production of strain-specific EPS with a wide variety of biological activities [110] that include, among others, the antioxidant, immunomodulatory, cholesterol lowering, antimicrobial and prebiotic effects (Figure 3). The main activities will be reviewed in the following sections.

### 4.1. EPS and Antioxidant Properties

Nowadays, there is an increasing scientific interest in the effects of reactive oxygen species (ROS) accumulation that cause in vivo oxidative stress conditions, leading to many chronic inflammatory conditions in the gut, as well as degenerative systemic diseases (atherosclerosis, diabetes, Alzheimer’s disease, inflammatory bowel disease, cardiovascular diseases, aging, and cancer) [111,112]. ROS, such as superoxide (^−^O_2_), hydroxyl radical (OH^•^), nitric oxide (NO) and peroxynitrite (ONOO^−^), are natural by-products of human cells which can cause serious damages to the cellular biomolecules, such as lipids, proteins and nucleic acids, thus a correct redox homeostasis is fundamental for maintaining a healthy cell metabolism and functions.

Currently, antioxidants from natural sources, including food-associated LAB and probiotics, have been investigated as dietary interventions to face ROS overproduction and accumulation [113,114]. As experimental data evidence, a higher intake of antioxidant molecules is associated with lower occurrence of human diseases [111,112]. 

The antioxidant role of EPS isolated from LAB strains, mainly from food origin, has been extensively investigated in in vitro systems. In general, the mechanisms by which bacterial EPS can exert antioxidant activity may include degradation of superoxide anion and hydrogen peroxide through ROS scavenging activity, inhibition of lipid peroxidation, reduction of metal ion chelating activity as well as up-regulation of enzymatic and non-enzymatic antioxidant activities [115,116,117]. 

A study carried out by Sengül et al. [118] reported that a high EPS-producing *Lb. delbrueckii* ssp. *bulgaricus* B3 strain exhibited higher antioxidant and metal ion chelating activities than *Lb. delbrueckii* ssp. *bulgaricus* A13, a low EPS-producing strain. More recently, other studies confirmed the antioxidant potential of EPS produced during milk fermentation, and in particular those from *Lb. delbrueckii ssp. bulgaricus* SRFM-1 [44].

EPS isolated from *Lpb. plantarum* C88 was found to possess antioxidant activity by scavenging ROS, upregulating of enzymatic- and non-enzymatic- antioxidant activities, and inhibiting lipid peroxidation [115]. In addition, EPS isolated from *Lcb. paracasei* ssp. *paracasei* NTU 101 and *Lpb. plantarum* 102 [119], *Lcb. rhamnosus* E/N [120], *Lpb. plantarum* C88 [115], *Lpb. plantarum* LP6 [121], *Lb. helveticus* MB2-1 [122], *Lpb. plantarum* BR2 [31], *Lactobacillus* spp. Ca6 [117] and *Lb. gasseri* FR4 [123] exhibited free radical scavenging activity, lipid peroxidation inhibition, β-Carotene bleaching reducing power, reducing and metal ion chelating activities in in vitro systems, reinforcing the potential use of LAB-derived EPS as naturally produced antioxidant food additives. Moreover, it has been observed that some EPS produced from different LAB species with antioxidant activity have also shown the ability to exert other beneficial effects such as immunomodulatory and anti-inflammatory properties, suggesting a potential correlation [109,119,124,125,126].

Enhanced activities of superoxide dismutase, glutathione peroxidase and catalase in murine hepatocytes and erythrocytes, as well as decreased levels of malondialdehyde by EPS, have been reported [115,127,128]. More recently, the antioxidant activity of EPS is also being confirmed by different in vivo studies [109,129,130]. In particular, a high dose of EPS (50 mg/kg per day) from *Lpb. plantarum* YW11 successfully ameliorate oxidative stress phenotypes in the aging mice model with a beneficial modulation of key *Phylum* in gut microbiota [129]. A similar result in the aging mice model has been found after the intake of EPS-1 produced by *Lb. helveticus* KLDS1.8701 that significantly alleviated liver injury and oxidative stress, together with a decrease in oxidative-stress related bacteria in the gut microbiota, confirming the correlation of antioxidant activity of EPS with gut microbiota modulation [130]. 

Although in vitro and in vivo studies conducted so far provide supportive and promising evidences for the use of EPS as natural strategy to counteract oxidative and inflammatory-related stress, future investigations should be carried out to elucidate the molecular mechanisms of EPS antioxidant activity, that are still not entirely understood.

### 4.2. EPS and Cholesterol-Lowering Effect

Cardiovascular disease (CVD) is one of the leading causes of death in Western societies, and hypertension and hypercholesterolemia are recognized as the two major risk factors [131]. Lifestyle changes, including dietary modifications, have a significant effect on the management of CVDs but adherence to low cholesterol and low-fat diets is, in actuality, not easy to maintain for a long-term period, leading to inefficacy over time [132]. On the other hand, pharmacological approaches, generally cholesterol-lowering drugs (i.e., statins), are still applied, despite the side effects. Recently, among dietary interventions, fermented dairy foods and/or probiotics, as well as specific microbial molecules such as EPS, have been shown a potential role in CVDs management for their lowering-cholesterol activity [133]. 

The administration of EPS produced by LAB has shown to be promising in the mitigation of CVD-related complications. For instance, EPS, produced by *Lb. lactis* ssp. *cremoris* SBT0495 in fermented milk, exhibited a positive effect on reducing cholesterol when fed to experimental hypercholesterolemic rats [134]. Significant improvements of HDL cholesterol and HDL to total cholesterol ratio were also observed when compared to the control group fed with milk fermented with a non-EPS-producing strain. Additionally, when kefiran EPS from *Lb. kefiranofaciens* WT-2B was fed to spontaneously hypertensive stroke-prone rats, significantly lower blood pressure and lower levels of cholesterol and triglycerides, both in serum and the liver, were observed compared to untreated rats [30]. Moreover, administration of 15 mg/kg body weight of EPS fractions from *Lcb. casei* LC2W in spontaneously hypertensive rats resulted in a moderate reduction in blood pressure [135]. 

A study by Tok and Aslim [131] suggested that high EPS-producing *Lb. delbrueckii* ssp. *bulgaricus* strains were more potent to sequester cholesterol from the medium in vitro compared to low EPS producer strains. Similarly, in an in vitro assay, EPS from *Lpb. plantarum* RJF4 (from rotten jack fruit) was able to reduce cholesterol level by 42.24% [125], and similarly, fruit-associated *Lpb. plantarum* BR2 by 45% [31]. Recently, EPS from *Lcb. paracasei* M7 showed higher in vitro cholesterol-lowering activity (70.78%) [133]. 

Moreover, in an in vivo assay, EPS isolated from *Lb. kefiranofaciens* was found to possess superior cholesterol-lowering properties by decreasing the level of extremely low-density lipoprotein cholesterol [136]. 

The exact mechanism behind cholesterol-lowering activity is still to be understood and may likely be multifactorial. It has been suggested that the EPS from bacteria can act in a similar way to dietary fibers that adsorb the cholesterol [137]. However, mechanisms associated with increased secretion of bile acids and reduced absorption of cholesterol by EPS were also proposed [138,139,140]. It has been also hypothesized that the bacterial cells can remove cholesterol from circulation by passively adsorbing it onto their membranes [99] or by assimilating it during cell growth [141].

Another lowering-cholesterol mechanism by LAB is through bile salt hydrolase activity (BSH), which decreases the resorption of bile acids by bile acid deconjugation and increases the elimination of the deconjugated bile acids in the enterohepatic circulation, leading to new bile acids synthesis from cholesterol in the liver [142]. *Lpb. plantarum* BSH activity has been also associated with the prevention of CVDs in human clinical trials [132]. 

The evidences described in this section, in particular the promising in vivo data, suggest that EPS-producing LAB and/or crude EPS, as well as the fermented foods containing them, could potentially offer a natural dietary alternative to the synthetic statins and bile acid sequestrants, which can cause undesirable side effects, for the management of CVDs and related diseases.

### 4.3. EPS and Antibacterial Activity

LAB-derived EPS have also claimed their antibacterial activity towards gram-positive and gram-negative food pathogens and/or for their competitive exclusion of pathogenic bacteria in the gastrointestinal tract (GI). 

For example, *Salmonella enterica* ATCC 43972 and *Micrococcus luteus* were effectively inhibited by EPS-Ca6 isolated from *Lactobacillus* sp. Ca6 [117]. Additionally, EPS-DN1 isolated from *Lb. kefiranofaciens* DN1 exhibited notable bactericidal activity against pathogenic bacteria such as *Listeria monocytogenes* and *Salmonella* *enteritidis* in a dose-dependent manner [143]. EPS-C70 produced by *Lpb. plantarum* isolated from camel milk showed a good inhibitory activity towards food-borne pathogens, including *Staphylococcus aureus* and *E. coli* [144].

Furthermore, EPS produced by *Lb. johnsonii* FI9785 may favor the colonization of probiotic bacteria in the host gastrointestinal tract by replacing pathogenic bacteria in a competitive inhibition mode [145]. 

A possible in vitro mechanism behind the antimicrobial activity of LAB-derived EPS may be related to their ability to interfere with the formation of pathogens biofilms by disrupting cellular integrity and communications [146,147]. The capability to form biofilm enhances the environmental survival and persistence of pathogenic bacteria, increasing the incidence of chronic and recurrent infections as well as raising antibiotic resistance [148], being responsible for safety concerns in both the food industry and in a medical environment. 

Antibiofilm activity of EPS from different LAB has been reported against pathogenic bacteria such as *E. faecalis*, *B. cereus* and *P. aeruginosa* [149]. In particular, a high antibiofilm activity has been found for EPS synthetized by *Leuconostoc citreum* isolated from sausages [150]. Recently, EPS from *Lcb. paracasei* M7 has been shown a broad spectrum of biofilm inhibition: *Enterococcus faecalis* (64.27%), *Bacillus subtilis* (63.84%), *Bacillus cereus*, (62.89%), *S. aureus* (61.45%), *Klebsiella* sp. (59.42%), *P. aeruginosa* (58.88%) [133], while dextran by probiotic *W. confusa* from Romanian yogurt showed a high biofilm inhibition percentage (70%) towards *Candida albicans* [151].

It has been suggested that EPS can inhibit the initial auto-aggregation of biofilm-forming bacteria by either interrupting communications among cells or by weakening cell membranes [152], and this ability is considered to be dose-dependent [153]. On the other side, it has been suggested that EPS may enhance the possibility of LAB colonization in the gut and its resistance to pathogenic bacteria. However, this speculation is yet to be tested related to LAB-derived EPS. Recently, it has been reported that EPS secreted by *Lim. fermentum* UCO-979C was able to reduce *H. pylori* adhesion (~30%) in both in vitro and in vivo studies, being also involved in ameliorating the immune response in *H. pylori* infections [154].

Chronic infections and antibiotic resistance are serious challenges for both food safety and population health. Thus, the potential of LAB-derived EPS to counteract biofilm formation could be used to lower infectious disease incidence caused by biofilm-producing pathogenic bacteria, as well as represent a promising alternative antimicrobial strategy as a growing number of evidence shows resistance to conventional antibiotics by pathogens.

### 4.4. Prebiotic Effects of EPS

Nowadays, the human gut microbiota, one of the most populated microbial communities ever known, has been considered fundamental for host health. Microbial populations harboring the GI environment act as metabolic and immunologic mediators, being directly involved in the maintenance of gut homeostasis [155]. It is well-known that a wide variety of factors can influence the composition of GI microbiota, including host endogenous (i.e., genetics or physiological status) and environmental factors. Among the latter, the diet represents one of the major environmental determinants of a healthy microbiota, in terms of composition and metabolic activity. In this perspective, a diet rich in dietary fibers and prebiotics is gaining attention as a strategy to positively modulate the gut microbiota by selectively increasing the number of beneficial microorganisms at the expense of the proliferation of harmful species, thus maintaining a correct balance in the gut ecosystem [156]. 

Prebiotics, were initially defined as “non-digestible food ingredients that beneficially affect the host by selectively stimulating the growth and/or activity of one or a limited number of bacteria in the colon, and thus improve host health” [157], and this definition was recently modified by the International Association of Probiotics and Prebiotics (ISAPP) as “a substrate that is selectively utilized by host microorganisms conferring a health benefit”, enlarging the potential prebiotics effects also to extra-intestinal sites [158]. 

In order to be claimed as prebiotics, a compound needs to fulfill the following criteria: “(i) be resistant to gastric and intestinal digestion, (ii) not be absorbed in the small intestine, (iii) be fermented by the intestinal microbiota, and (iv) produce a beneficial effect on host health through the selective stimulation of microbial populations or their metabolic activities” [159].

Recently, EPS secreted by LAB have been claimed to have a potential prebiotic effect similar to diet-derived polysaccharides from plant origin [7,24,155,159]. In particular, it has been shown that the capacity to endure the harsh condition of the GI tract may vary among different EPS and is strictly dependent on their physicochemical properties [105], being ultimately strain-specific in their gut microbiota modulation ability [6]. 

Considering that, several studies have been focused on evaluating the EPS resistance to both gastric and intestinal conditions by in vitro mimicking digestion and putative EPS fermentation by bacteria belonging to intestinal microbiota species [160,161,162,163]. In general, the prebiotic effect of EPS consists of promoting the selective growth of beneficial and/or probiotic bacteria (mainly LAB and bifidobacteria) that are able to enzymatically digest EPS, while the inhibition of *Enterobacteriaceae* is one of the main desirable features.

EPS from *Lpb. plantarum* showed high resistance to the acidity of gastric environment with a notable bifidogenic effect by selectively improving the growth of bifidobacteria [161]. EPS from *Lpb. plantarum* DM5 have been shown to promote the growth of *Bifidobacterium infantis* and *Lb. acidophilus* in an in vitro study [162]. A similar bifidogenic effect has been also reported in a previous study examining the prebiotic effect of levan EPS from *Fructilactobacillus* (*Fr.*) *sanfranciscensis* [160].

Recently, a strong selective growth enhancement of bifidobacteria, lactobacilli and lactococci has been found by two different EPS fractions from *Lb. delbrueckii* ssp. *bulgaricus* SRFM-1 in an in vitro digestion and fermentation study, in which EPS also showed a high production of short-chain fatty acids (SCFA) after fermentation of human faecal samples [163]. Moreover, EPS from *Lb. delbrueckii* ssp. *bulgaricus* SRFM-1 showed the ability to promote the growth of *S. thermophilus* and an inhibitory effect on *E. coli* [164], suggesting that LAB-derived EPS could be good candidates to be used as prebiotics additives for functional foods.

However, some in vivo studies reported controversial effect of EPS (β-D-glucan) from *Pediococcus parvulus.* In particular, β-D-glucan produced from *P. parvulus* 2.6 has been shown to enhance the growth and increase adherence to intestinal cells of different probiotic strains in vitro [165], but no specific physiological responses correlated to *P. parvulus* 2.6 have been found in an experimental model with mice [166].

The main challenge in determining the effectiveness of the prebiotics effect of EPS secreted by LAB, is that usually the EPS biodegradability and its modulating effect are mainly investigated by in vitro assays that mimic gastrointestinal conditions during digestion, but often these conditions do not consider the effective interaction among the bacterial population in the gut as well as host-microbe interactions [24]. In this perspective, the use of the novel gut fermentation system, in which it is possible to monitor both the EPS digestion and the fecal microbiota changes, can be a useful tool to examine more deeply the potential prebiotic effect, even though the in vivo conditions are hard to entirely achieved [159]. Due to the complexity of metabolic activity of microbes in the gut, a possible correlation between EPS and other bioactive molecules (i.e., SCFA) secreted by some LAB species (*Lb. delbrueckii* subsp. *bulgaricus*, *S, salivarus* subsp. *thermophilus*, *Pediococcus damnosus* and *Lim. reuteri*) should also be taken into account [167]. 

It has been supposed that, after EPS digestion by intestinal bacteria, the residual oligo- and monosaccharides can be used as a carbon source by many gut inhabitants that will produce mainly SCFA and lactic acid as secondary metabolites, which consequently can be reused by cross-feeding mechanisms and/or can have other beneficial effects through the host–microbe interactions [159]. The most innovative -omics technologies have been also applied to better-elucidated microbiota variations induced by prebiotic substances [168,169]. 

This state-of-the-art application highlights that there is a lack of in vivo studies and no human intervention studies have been performed to date, thus demonstrating that the effectiveness of prebiotic effects of bacterial EPS in conferring health benefits through a selectively modulation of gut microbial populations still remains an open challenge.

## 5. Application of EPS in Dairy Industry

The industrial applications of EPS-producing LAB are gaining a growing interest because of their positive impact as thickening and structuring agents on rheology, texture and mouth-feel properties [170]. They are widely applied in fermented dairy products such as yogurt, cheese and fermented milks, as well as in dairy-based desserts. In fact, the in situ production of EPS is likely to have the ability to limit the use of added chemicals and stabilizers frequently applied to enhance the textural properties of fermented milk products [85]. A broad number of EPS-producing LAB species are currently included in dairy product formulations, and several studies on the effects of these EPS-producing strains on the textural properties and stability of fermented dairy products are available (Table 4).

From the data reported in Table 4, it could be evidenced that microbial EPS play an important role in defining physicochemical (viscosifying, stabilizing or water-binding capacities) and sensorial (palatability) characteristics of final products. The use of EPS-producing LAB for yogurt and fermented milks and beverages allows to reduce the quantity of added milk solids, and to improve some qualitative attributes such as viscosity, texture and stability, and avoid syneresis [68]. In yogurt industrial production, the use of EPS-producing *S. thermophilus* and *Lb. delbrueckii* subsp. *bulgaricus* strains, due to their synergistic action, leads to a decrease in syneresis and higher viscosity, as well as a smoother and creamy texture, offering a valid alternative to the use of stabilizers (gums, starches, gelatin) and/or of some dry dairy ingredients (i.e., whey protein, skim milk powder, sodium- or calcium caseinates) generally used to obtain a desirable yogurt texture [83,173,174,179]. A successful and positive interaction between other EPS-producing species, such as *Lb. kefiranofaciens,* with the traditional yogurt starter cultures in improving viscosity and avoiding syneresis of yogurt has also been shown [84]. EPS in situ production is exploited in traditional fermented beverages such as kefir, dahi and pulque. *Lb. kefiranofaciens* is the best producer of kefiran, a water-soluble EPS produced in kefir grains. It is a branched glucogalactan composed of hexa- or heptasaccharide repeating structure with almost equal amounts of glucose and galactose [180].

*Leuconostoc mesenteroides* is the main microorganism involved in pulque fermentation. The strain IBT-PQ isolated from pulque produced a soluble linear dextran with glucose molecules linked by α-(1,6) bonds with branching from α-(1,3) bonds in a 4:1 ratio, respectively [181]. Some EPS-producing LAB strains, such as *Lact. lactis* subsp. *lactis* PM23, *S. thermophilus* ST and *Lact. lactis* NCDC 191 have been applied for improving texture and flavour of dahi, a traditional yogurt very popular in South Asia, usually made through the fermentation of buffalo, cow or goat milk [182].

EPS-producing strains are also applied to improve the technological traits of low-fat cheeses [183]. The use of the EPS-producing *S. thermophilus* strain allowed to improve the sensory attributes of a low-fat Caciotta-type cheese [171]. Moreover, the use of an EPS-producing *Lact. lactis* strain for reduced-fat Cheddar cheese induced an increase in moisture content, water activity and water desorption rate [184]. The inclusion of EPS-producing cultures in an Egyptian cheese Karish improved its acceptability and creaminess [185]. 

In general, the application of EPS-producing strains provides a positive effect in reduced-fat cheeses since they increase the moisture, reduce the rigidity of the protein network and increase viscosity of the serum phase [183]. However, one of the major limiting factors for the use of EPS-producing LAB for industrial applications is the relatively low in situ production of EPS by LAB strains that remain the major challenge to face in order to widen the use of EPS as thickeners and/or texture-forming natural additives in the dairy industry. Moreover, no standardized protocols are available for an EPS-producing starter cultures selection. In fact, the absence of a clear structure–process–function relationship is a problem for the screening of tailored EPS-producing starter cultures. In fact, EPS production is influenced by food matrix composition and processing conditions. Another aspect which makes the assessment of a standardized protocol difficult is that the EPS should be produced in an amount at which functionality is guaranteed. This is particularly difficult when raw materials are subject to variations and thus cannot be standardized. In this sense, genetic engineering may be useful to drive the production of specific EPS with interesting rheological or biological characteristics.

## 6. Conclusions

In the glossary to the report of the Dahlem Workshop on Structure and Function of Biofilms 1988, EPS are defined as “organic polymers of microbial origin which in biofilm systems are frequently responsible for binding cells and other particulate materials together (cohesion) and to the substratum (adhesion)” [186]. Starting from that point, EPS have gained scientific interest. 

The majority of studies are focused on the isolation and characterization of microbial EPS, owing to their importance in industrial applications. However, their use in food industries is still limited because of the high production costs. The possibility to reduce costs and improve yield—using waste biomass such as molasses—are the main challenges in this field. Improvement of the fermentation conditions and the downstream steps for EPS recovery are essential for a market massive use of microbial polysaccharides. Genetic and metabolic engineering will be also useful for the widespread use of these biopolymers of microbial origin. 

The research interest in EPS is increasing also because of its health benefits. Many in vitro studies have reported antioxidant, cholesterol-lowering, antimicrobial, anti-biofilm as well as prebiotics and immunomodulatory activities of EPS. However, in vivo studies are still limited, and there is much more to further investigate to increase the EPS yield to enlarge industrial applications, as well as more in vivo and clinical studies needed to validate the entire potential of EPS to be used as functional adjuncts.

## Figures and Tables

**Table 1:** Industrial applications and health properties of some HoPS and HePS.

EPS	Producers	Biological Properties
**HoPS**		
Dextran (glucose)	*Leuconostoc mesenteroides, Lactobacillus reuteri, Lacticaseibacillus casei, Latilactobacillus sakei, Limosilactobacillus fermentum, Lentilactobacillus parabuchneri*	Food industry: emulsifier and stabilizer, improvement of softness, crumb texture, loaf volume in bakery products, improvement of moisture retention and viscosity in confectionary
Reuteran (glucose)	*Lactobacillus reuteri*	Bakery industry
Levan (fructose)	*Bacillus subtilis, Streptococcus salivarius, Streptococcus mutans*	Health benefits: prebiotic activity, antitumor property, hypocholesterolemic agent Food industry: bio-thickener
Inulin-type (fructose)	*Streptococcus mutans, Lactobacillus reuteri*	Health benefits: prebiotic activity Food industry: sugar and fat replacer, texture modifier in low-fat dairy products enhancer of creaminess
Alternan (glucose)	*L. mesenteroides, Leuconostoc citreum*	Food industry: Sweetener in confectionary, stabilizer, emulsifier and prebiotics
Curdlan (glucose)	*Alcaligenes faecalis, Rhizobium meliloti, Agrobacterium radiobacter*	Starter culture, gelling agent, immoblization matrix
**HePS**		
Glucose and galactose	*Lactiplantibacillus plantarum, Streptococcus thermophilus, Enterococcus faecium*	Health benefits: immunomodulatory activity
Arabinose, mannose, glucose and galactose	*Lpb. plantarum*	Food industry: improvement of texture and rheological properties of various food stuffs
Glucose, arabinose, galactose, mannose and xylose	*Bacillus tequilensis*	Food industry: stabilizer and thickener
Glucose, mannose, galactose, rhamnose, and a small fraction fucose	*Lactobacillus gasseri*	Food industry: antioxidant agent, viscosifying agent and antimicrobial agent
Arabinose, rhamnose, fucose, xylose, mannose, fructose, galactose and glucose	*Lpb. plantarum*	Food industry: natural antioxidant or functional additive

**Table 2:** Exopolysaccharide produced by some LAB strains growing in different media, carbon source and its monosaccharide composition.

Genus	Strains	EPS Yield (mg/L)	Culture Media	Carbon Source in Media	Monosaccharide Composition	Reference
*Streptococcus*						
	*S. thermophilus* DGCC7919	404	Whey permeate	Lactose	Glucose, Galactose, Rhamnose, Mannose	[33]
	*S. thermophilus* ASCC 1275	163–430	M17	Glucose or lactose or sucrose	Glucose, Galactose	[34]
	*S. thermophilus* S-3	100	Skim milk	Lactose	Glucose, Galactose	[35]
	*S. thermophilus* CC30	1950	Skim milk	Lactose	Glucose, Galactose	[36]
	*S. thermophilus* 05-34	55–120	Skim milk	Glucose or Galactose or Lactose or Sucrose or Fructose	Glucose, Galactose	[37]
	*S. thermophilus* GST-6	Not reported	Skim milk	Lactose	Glucose, Galactose	[38]
	*S. thermophilus* ST1	136	Skim milk	Lactose and sucrose	Glucose, Galactose	[39]
	*S. thermophilus* CRL804	166	Skim milk	Lactose	Galactose, Rhamnose	[12]
	*S. thermophilus* SY89, SY102, IMDO1, IMDO2, IMDO3, NCFB 859	Not reported	Skim milk	Lactose	Glucose, Galactose	[40]
	*S. thermophilus* NCFB 2393	300	Skim milk	Lactose	Glucose, Galactose, Rhamnose	[41]
*Lactobacillus*						
	*Lb. delbrueckii* subsp. *bulgaricus*	5570.34–5910.62	Modified Skim milk	Glucose	Glucose, Galactose, Rhamnose, Ribose, Mannose, Xylose, Arabinose, Fructose	[42]
	*Lb. delbrueckii* subsp. *bulgaricus* 147	960	Skim milk	Lactose	Glucose, Galactose, Rhamnose, Ribose, Mannose	[43]
	*Lb. delbrueckii* subsp. *bulgaricus* 2214	1880	Skim milk	Lactose	Glucose, Rhamnose, Mannose	[43]
	*Lb. delbrueckii* subsp. *bulgaricus* B3	449	MRS	Glucose	Glucose, Fructose, Mannose	[27]
	*Lb. delbrueckii* subsp. *bulgaricus* SRFM-1	141.63	Milk	Lactose	Glucose, Galactose	[44]
	*Lb. delbrueckii* subsp. *bulgaricus* OLL1073R-1	1546	Skim milk	Lactose	Glucose, Galactose	[45]
	*Lb. delbrueckii* subsp. *bulgaricus*	190–740	Modified MRS	Lactose and Glucose	Not reported	[46]
	*Lb. delbrueckii* subsp. *bulgaricus* CRL 852, 865, 874	24–150	Skim milk	Lactose	Glucose, Galactose, Rhamnose	[12]
	*Lb. delbrueckii* subsp. *bulgaricus* LY03	Not reported	Skim milk	Lactose	Glucose, Galactose, Rhamnose	[40]
	*Lb. helveticus* LZ-R-5	128	Milk	Lactose	Glucose, Galactose	[47]
*Lactiplantibacillus*						
	*Lpb. plantarum* subsp. *plantarum* JLAU103	75	Modified MRS	Sorbitol	Glucose, Galactose, Rhamnose, Mannose, Xylose, Arabinose, Fructose, Fucose	[48]
	*Lpb. plantarum* subsp. *plantarum* GD2	397	MRS	Glucose	Glucose, Mannose, Arabinose	[27]
	*Lpb. plantarum* subsp. *plantarum* JLK0142	Not reported	Modified MRS	Sorbitol	Glucose, Galactose	[49]
	*Lpb. plantarum* subsp. *plantarum* C7	198–265	Modified MRS	Sucrose	Glucose, Mannose	[50]
*Limosilactobacillus*						
	*Lim. reuteri* L26 *and Lim. reuteri* DSM 17938	4300–5000	Modified MRS	Sucrose	Glucose	[32]
	*Lim. fermentum* YL-11	84.5	Skim milk	Lactose	Glucose, Galactose, Mannose, Arabinose	[26]
*Lacticaseibacillus*						
	*Lcb. rhamnosus* E9	298	MRS	Glucose	Glucose, Mannose, Arabinose	[27]
	*Lcb. rhamnosus* RW-9595M	931–1275	MRS	Glucose	Glucose, Galactose, Rhamnose	[51]
	*Lcb. rhamnosus* R	438–601	MRS	Glucose or Lactose	Glucose, Galactose, Rhamnose	[51]
*Levilactobacillus*						
	*Lev. brevis* LB63	347	MRS	Glucose	Glucose, Mannose, Arabinose	[27]
*Lactococcus*						
	*Lact. lactis* LL-2A	354	Whey permeate	Lactose	Glucose, Galactose, Mannose	[33]
	*Lact. lactis* SLT10	280–336	Modified MRS	Sucrose	Glucose, Mannose, Rhamnose	[50]
	*Lact. lactis* subsp. *cremoris* JFR1	Not reported	Skim milk	Lactose	Glucose, Galactose, Rhamnose	[52]
	*Lact. lactis* subsp. *cremoris* 352	204	Skim milk	Lactose	Glucose, Galactose	[19]

**Table 3:** Main factors influencing EPS production by LAB strains.

Main Influencing Factors	Example	Reference
Microbial factors		
EPS producers	Species/strains	[8,12,24,35,53]
Bacterial growth	Exponential/stationary phase	[38,60,68,82,83]
Microbial interactions	Synergistic/antagonist effects in bacterial mixture	[71,84]
Medium composition		
Carbon source	Glucose/Sucrose/Lactose etc.	[34,37,39,55,56,60,64,82]
Nitrogen source	Organic/inorganic nitrogen	[37,57,60,68,82]
Physicochemical parameters		
pH	pH of medium and its variations	[37,54,57,60,68,82]
Temperature	Optimal/sub-optimal temperature	[37,39,57,59,60,64,67,68,69,82]
Oxygen rate	Aerobiosis/anaerobiosis	[24,60,67,85]
Incubation time	Prolonged incubation time	[37,39,55,57,64,66,68,82]

**Table 4:** EPS-producing strains in fermented milk products and their effects on techno-functional properties.

EPS-Producers	Foods	Main Effects	Reference
*S. thermophilus*	Yogurt drink (Ayran)	Increased apparent viscosity	[107]
*S. thermophilus*	Low-fat Italian Cacciotta type cheese	Improved taste, flavor and chew ability	[171]
*S. thermophilus* ST-5581, ST-4239 and ST-PH *Lact.lactis* subsp. *cremoris* JFR1	Fermented milk	Low syneresis, increased viscosity, lowered incidence of colon tumor and activity of cyclooxygenase-2 enzyme	[172]
*S.thermophilus* ASCC 1275	Set and stirred yogurt	Decreased firmness and syneresis	[173]
*S. thermophilus* zlw TM11	Yogurt	Improved yogurt texture and lower separation of whey	[174]
*Lb. delbrueckii* subsp. *bulgaricus* 147 and 2214	Fermented milk	Improved viscosity, low syneresis and increased water holding capacity	[43]
*Lb. delbrueckii* subsp. *bulgaricus* CNRZ 1187 and 416	Fermented milk	Improved viscosity	[175]
*Lpb. plantarum* KX881772 and KX881779	Low-fat Akawi cheese	Improved water holding capacity, improved elastic structure, reduced hardness and adhesiveness, higher scores in appearance and overall quality	[176]
*Lim. fermentum* Lf2	Non-fat yogurt	Creamy consistency, increased hardness, improved water holding capacity and low syneresis	[177]
*Lim. mucosae* DPC 6426	Low-fat yogurt	Improved viscosity and reduced syneresis	[178]
*Lcb. rhamnosus* JAAS8	Yogurt	Increased apparent viscosity and improved water holding capacity	[83]

## References

[1] Sanlibaba P., Cakmak G.A. (2016). Exopolysaccharides Production by Lactic Acid Bacteria. Appl. Microbiol. Open Access.

[2] Donot F., Fontana A., Baccou J.C., Schorr-Galindo S. (2012). Microbial Exopolysaccharides: Main Examples of Synthesis, Excretion, Genetics and Extraction. Carbohydr. Polym..

[3] Flemming H.-C. (2016). EPS—Then and Now. Microorganisms.

[4] Kumar A.S., Mody K., Jha B. (2007). Bacterial Exopolysaccharides—A Perception. J. Basic Microbiol..

[5] Galle S., Schwab C., Dal Bello F., Coffey A., Gänzle M.G., Arendt E.K. (2012). Influence of In-Situ Synthesized Exopolysaccharides on the Quality of Gluten-Free Sorghum Sourdough Bread. Int. J. Food Microbiol..

[6] Ryan P.M., Ross R.P., Fitzgerald G.F., Caplice N.M., Stanton C. (2015). Sugar-Coated: Exopolysaccharide Producing Lactic Acid Bacteria for Food and Human Health Applications. Food Funct..

[7] Caggianiello G., Kleerebezem M., Spano G. (2016). Exopolysaccharides Produced by Lactic Acid Bacteria: From Health-Promoting Benefits to Stress Tolerance Mechanisms. Appl. Microbiol. Biotechnol..

[8] Lynch K.M., Zannini E., Coffey A., Arendt E.K. (2018). Lactic Acid Bacteria Exopolysaccharides in Foods and Beverages: Isolation, Properties, Characterization, and Health Benefits. Annu. Rev. Food Sci. Technol..

[9] Zheng J., Wittouck S., Salvetti E., Franz C.M.A.P., Harris H.M.B., Mattarelli P., O’toole P.W., Pot B., Vandamme P., Walter J. (2020). A Taxonomic Note on the Genus *Lactobacillus*: Description of 23 Novel Genera, Emended Description of the Genus *Lactobacillus beijerinck* 1901, and Union of Lactobacillaceae and Leuconostocaceae. Int. J. Syst. Evol. Microbiol..

[10] Castro-Bravo N., Wells J.M., Margolles A., Ruas-Madiedo P. (2018). Interactions of Surface Exopolysaccharides from *Bifidobacterium* and *Lactobacillus* within the Intestinal Environment. Front. Microbiol..

[11] Van Kranenburg R., Vos H.R., Van Swam I.I., Kleerebezem M., De Vos W.M. (1999). Functional Analysis of Glycosyltransferase Genes from *Lactococcus lactis* and Other Gram-Positive Cocci: Complementation, Expression, and Diversity. J. Bacteriol..

[12] Mozzi F., Vaningelgem F., Hébert E.M., Van Der Meulen R., Moreno M.R.F., Font De Valdez G., De Vuyst L. (2006). Diversity of Heteropolysaccharide-Producing Lactic Acid Bacterium Strains and Their Biopolymers. Appl. Environ. Microbiol..

[13] Torino M.I., de Valdez G.F., Mozzi F. (2015). Biopolymers from Lactic Acid Bacteria. Novel Applications in Foods and Beverages. Front. Microbiol..

[14] Chapot-Chartier M.P., Kulakauskas S. (2014). Cell Wall Structure and Function in Lactic Acid Bacteria. Microb. Cell Fact..

[15] Leemhuis H., Pijning T., Dobruchowska J.M., van Leeuwen S.S., Kralj S., Dijkstra B.W., Dijkhuizen L. (2013). Glucansucrases: Three-Dimensional Structures, Reactions, Mechanism, α-Glucan Analysis and Their Implications in Biotechnology and Food Applications. J. Biotechnol..

[16] Zeidan A.A., Poulsen V.K., Janzen T., Buldo P., Derkx P.M.F., Øregaard G., Neves A.R. (2017). Polysaccharide Production by Lactic Acid Bacteria: From Genes to Industrial Applications. FEMS Microbiol. Rev..

[17] Deo D., Davray D., Kulkarni R. (2019). A Diverse Repertoire of Exopolysaccharide Biosynthesis Gene Clusters in *Lactobacillus* Revealed by Comparative Analysis in 106 Sequenced Genomes. Microorganisms.

[18] Remus D.M., van Kranenburg R., van Swam I.I., Taverne N., Bongers R.S., Wels M., Wells J.M., Bron P.A., Kleerebezem M. (2012). Impact of 4 *Lactobacillus plantarum* Capsular Polysaccharide Clusters on Surface Glycan Composition and Host Cell Signaling. Microb. Cell Fact..

[19] Knoshaug E.P., Ahlgren J.A., Trempy J.E. (2007). Exopolysaccharide Expression in Lactococcus lactis subsp. cremoris Ropy 352: Evidence for Novel Gene Organization. Appl. Environ. Microbiol..

[20] Goh Y.J., Goin C., O’Flaherty S., Altermann E., Hutkins R. (2011). Specialized Adaptation of a Lactic Acid Bacterium to the Milk Environment: The Comparative Genomics of *Streptococcus thermophilus* LMD-9. Microb. Cell Fact..

[21] Mistou M.Y., Sutcliffe I.C., Van Sorge N.M. (2016). Bacterial Glycobiology: Rhamnose-Containing Cell Wall Polysaccharides in Gram-Positive Bacteria. FEMS Microbiol. Rev..

[22] Thevenard B., Besset C., Choinard S., Fourcassié P., Boyaval P., Monnet V., Rul F. (2014). Response of *S. thermophilus* LMD-9 to Bacitracin: Involvement of a BceRS/AB-like Module and of the Rhamnose-Glucose Polysaccharide Synthesis Pathway. Int. J. Food Microbiol..

[23] Mahony J., Kot W., Murphy J., Ainsworth S., Neve H., Hansen L.H., Heller K.J., Sørensen S.J., Hammer K., Cambillau C. (2013). Investigation of the Relationship between *Lactococcal* Host Cell Wall Polysaccharide Genotype and 936 Phage Receptor Binding Protein Phylogeny. Appl. Environ. Microbiol..

[24] Badel S., Bernardi T., Michaud P. (2011). New Perspectives for Lactobacilli Exopolysaccharides. Biotechnol. Adv..

[25] Khalil E.S., Manap M.Y.A., Mustafa S., Alhelli A.M., Shokryazdan P. (2018). Probiotic Properties of Exopolysaccharide-Producing *Lactobacillus* Strains Isolated from Tempoyak. Molecules.

[26] Wei Y., Li F., Li L., Huang L., Li Q. (2019). Genetic and Biochemical Characterization of an Exopolysaccharide With in Vitro Antitumoral Activity Produced by *Lactobacillus fermentum* YL-11. Front. Microbiol..

[27] Tukenmez U., Aktas B., Aslim B., Yavuz S. (2019). The Relationship between the Structural Characteristics of Lactobacilli-EPS and Its Ability to Induce Apoptosis in Colon Cancer Cells in Vitro. Sci. Rep..

[28] Tsuda H., Miyamoto T. (2010). Production of Exopolysaccharide by *Lactobacillus plantarum* and the Prebiotic Activity of the Exopolysaccharide. Food Sci. Technol. Res..

[29] Macedo M.G., Laporte M.F., Lacroix C. (2002). Quantification of Exopolysaccharide, Lactic Acid, and Lactose Concentrations in Culture Broth by near-Infrared Spectroscopy. J. Agric. Food Chem..

[30] Maeda H., Zhu X., Suzuki S., Suzuki K., Kitamura S. (2004). Structural Characterization and Biological Activities of an Exopolysaccharide Kefiran Produced by *Lactobacillus kefiranofaciens* WT-2B T. J. Agric. Food Chem..

[31] Sasikumar K., Kozhummal Vaikkath D., Devendra L., Nampoothiri K.M. (2017). An Exopolysaccharide (EPS) from a *Lactobacillus plantarum* BR2 with Potential Benefits for Making Functional Foods. Bioresour. Technol..

[32] Kšonžeková P., Bystrický P., Vlčková S., Pätoprstý V., Pulzová L., Mudroňová D., Kubašková T., Csank T., Tkáčiková L. (2016). Exopolysaccharides of *Lactobacillus reuteri*: Their Influence on Adherence of E. Coli to Epithelial Cells and Inflammatory Response. Carbohydr. Polym..

[33] Nachtigall C., Surber G., Herbi F., Wefers D., Jaros D., Rohm H. (2020). Production and Molecular Structure of Heteropolysaccharides from Two Lactic Acid Bacteria. Carbohydr. Polym..

[34] Padmanabhan A., Tong Y., Wu Q., Zhang J., Shah N.P. (2018). Transcriptomic Insights into the Growth Phase- and Sugar-Associated Changes in the Exopolysaccharide Production of a High EPS-Producing *Streptococcus thermophilus* ASCC 1275. Front. Microbiol..

[35] Xu Z., Guo Q., Zhang H., Wu Y., Hang X., Ai L. (2018). Exopolysaccharide Produced by *Streptococcus thermophiles* S-3: Molecular, Partial Structural and Rheological Properties. Carbohydr. Polym..

[36] Kanamarlapudi S.L.R.K., Muddada S. (2017). Characterization of Exopolysaccharide Produced by *Streptococcus thermophilus* CC30. BioMed Res. Int..

[37] Li D., Li J., Zhao F., Wang G., Qin Q., Hao Y. (2016). The Influence of Fermentation Condition on Production and Molecular Mass of EPS Produced by *Streptococcus thermophilus* 05-34 in Milk-Based Medium. Food Chem..

[38] Zhang J., Cao Y., Wang J., Guo X., Zheng Y., Zhao W., Mei X., Guo T., Yang Z. (2016). Physicochemical Characteristics and Bioactivities of the Exopolysaccharide and Its Sulphated Polymer from *Streptococcus thermophilus* GST-6. Carbohydr. Polym..

[39] Zhang T., Zhang C., Li S., Zhang Y., Yang Z. (2011). Growth and Exopolysaccharide Production by *Streptococcus thermophilus* ST1 in Skim Milk. Braz. J. Microbiol..

[40] Marshall V.M., Laws A.P., Gu Y., Levander F., Rådström P., De Vuyst L., Degeest B., Vaningelgem F., Dunn H., Elvin M. (2001). Exopolysaccharide-Producing Strains of Thermophilic Lactic Acid Bacteria Cluster into Groups According to Their EPS Structure. Lett. Appl. Microbiol..

[41] Almiron-Roig E., Mulholland F., Gasson M.J., Griffin A.M. (2000). The Complete Cps Gene Cluster from *Streptococcus thermophilus* NCFB 2393 Involved in the Biosynthesis of a New Exopolysaccharide. Microbiology.

[42] Adebayo-Tayo B., Fashogbon R. (2020). In Vitro Antioxidant, Antibacterial, in Vivo Immunomodulatory, Antitumor and Hematological Potential of Exopolysaccharide Produced by Wild Type and Mutant *Lactobacillus delbureckii* subsp. bulgaricus. Heliyon.

[43] Bancalari E., Alinovi M., Bottari B., Caligiani A., Mucchetti G., Gatti M. (2020). Ability of a Wild *Weissella* Strain to Modify Viscosity of Fermented Milk. Front. Microbiol..

[44] Tang W., Dong M., Wang W., Han S., Rui X., Chen X., Jiang M., Zhang Q., Wu J., Li W. (2017). Structural Characterization and Antioxidant Property of Released Exopolysaccharides from Lactobacillus delbrueckii ssp. bulgaricus SRFM-1. Carbohydr. Polym..

[45] Van Calsteren M.R., Gagnon F., Nishimura J., Makino S. (2015). Structure Determination of the Neutral Exopolysaccharide Produced by *Lactobacillus delbrueckii* subsp. *bulgaricus* OLL1073R-1. Carbohydr. Res..

[46] Abdellah M., Ahcne H., Benalia Y., Saad B., Abdelmalek B. (2015). Evaluation of Biofilm Formation by Exopolysaccharide-Producer Strains of Thermophilic Lactic Acid Bacteria Isolated from Algerian Camel Milk. Emir. J. Food Agric..

[47] You X., Li Z., Ma K., Zhang C., Chen X., Wang G., Yang L., Dong M., Rui X., Zhang Q. (2020). Structural Characterization and Immunomodulatory Activity of an Exopolysaccharide Produced by *Lactobacillus helveticus* LZ-R-5. Carbohydr. Polym..

[48] Min W.H., Fang X.B., Wu T., Fang L., Liu C.L., Wang J. (2019). Characterization and Antioxidant Activity of an Acidic Exopolysaccharide from *Lactobacillus plantarum* JLAU103. J. Biosci. Bioeng..

[49] Wang J., Wu T., Fang X., Min W., Yang Z. (2018). Characterization and Immunomodulatory Activity of an Exopolysaccharide Produced by *Lactobacillus plantarum* JLK0142 Isolated from Fermented Dairy Tofu. Int. J. Biol. Macromol..

[50] Ziadi M., Bouzaiene T., M’Hir S., Zaafouri K., Mokhtar F., Hamdi M., Boisset-Helbert C. (2018). Evaluation of the Efficiency of Ethanol Precipitation and Ultrafiltration on the Purification and Characteristics of Exopolysaccharides Produced by Three Lactic Acid Bacteria. BioMed Res. Int..

[51] Van Calsteren M.R., Pau-Roblot C., Bégin A., Roy D. (2002). Structure Determination of the Exopolysaccharide Produced by *Lactobacillus rhamnosus* Strains RW-9595M and R. Biochem. J..

[52] Ayala-Hernández I., Hassan A., Goff H.D., Mira de Orduña R., Corredig M. (2008). Production, Isolation and Characterization of Exopolysaccharides Produced by Lactococcus lactis subsp. cremoris JFR1 and Their Interaction with Milk Proteins: Effect of PH and Media Composition. Int. Dairy J..

[53] Leroy F., De Vuyst L. (2016). Advances in Production and Simplified Methods for Recovery and Quantification of Exopolysaccharides for Applications in Food and Health. J. Dairy Sci..

[54] Jolly L., Vincent S.J.F., Duboc P., Neeser J.R. (2002). Exploiting Exopolysaccharides from Lactic Acid Bacteria. Antonie van Leeuwenhoek Int. J. Gen. Mol. Microbiol..

[55] Cirrincione S., Breuer Y., Mangiapane E., Mazzoli R., Pessione E. (2018). ’Ropy’ Phenotype, Exopolysaccharides and Metabolism: Study on Food Isolated Potential Probiotics LAB. Microbiol. Res..

[56] Schwab C., Mastrangelo M., Corsetti A., Gänzle M. (2008). Formation of Oligosaccharides and Polysaccharides by *Lactobacillus reuteri* LTH5448 and *Weissella cibaria* 10M in Sorghum Sourdoughs. Cereal Chem..

[57] Vaningelgem F., Zamfir M., Mozzi F., Adriany T., Vancanneyt M., Swings J., De Vuyst L. (2004). Biodiversity of Exopolysaccharides Produced by *Streptococcus thermophilus* Strains Is Reflected in Their Production and Their Molecular and Functional Characteristics. Appl. Environ. Microbiol..

[58] Ruas-Madiedo P., De Los Reyes-Gavilán C.G. (2005). Invited Review: Methods for the Screening, Isolation, and Characterization of Exopolysaccharides Produced by Lactic Acid Bacteria. J. Dairy Sci..

[59] Rabha B., Nadra R.-S., Ahmed B. (2012). Effect of Some Fermentation Substrates and Growth Temperature on Exopolysaccharide Production by *Streptococcus thermophilus* BN1. Int. J. Biosci. Biochem. Bioinform..

[60] Petry S., Furlan S., Crepeau M.J., Cerning J., Desmazeaud M. (2000). Factors Affecting Exocellular Polysaccharide Production by Lactobacillus delbrueckii subsp. bulgaricus Grown in a Chemically Defined Medium. Appl. Environ. Microbiol..

[61] Wang M., Bi J. (2008). Modification of Characteristics of Kefiran by Changing the Carbon Source of *Lactobacillus kefiranofaciens*. J. Sci. Food Agric..

[62] Zajšek K., Goršek A., Kolar M. (2013). Cultivating Conditions Effects on Kefiran Production by the Mixed Culture of Lactic Acid Bacteria Imbedded within Kefir Grains. Food Chem..

[63] Degeest B., Janssens B., De Vuyst L. (2001). Exopolysaccharide (EPS) Biosynthesis by *Lactobacillus sakei* 0-1: Production Kinetics, Enzyme Activities and EPS Yields. J. Appl. Microbiol..

[64] Zhang Y., Li S., Zhang C., Luo Y., Zhang H., Yang Z. (2011). Growth and Exopolysaccharide Production by *Lactobacillus fermentum* F6 in Skim Milk. Afr. J. Biotechnol..

[65] Pham P.L., Dupont I., Roy D., Lapointe G., Cerning J. (2000). Production of Exopolysaccharide by *Lactobacillus rhamnosus* R and Analysis of Its Enzymatic Degradation during Prolonged Fermentation. Appl. Environ. Microbiol..

[66] Lin T.Y., Chien M.F.C. (2007). Exopolysaccharides Production as Affected by Lactic Acid Bacteria and Fermentation Time. Food Chem..

[67] Mende S., Krzyzanowski L., Weber J., Jaros D., Rohm H. (2012). Growth and Exopolysaccharide Yield of Lactobacillus delbrueckii ssp. bulgaricus DSM 20081 in Batch and Continuous Bioreactor Experiments at Constant PH. J. Biosci. Bioeng..

[68] De Vuyst L., De Vin F., Vaningelgem F., Degeest B. (2001). Recent Developments in the Biosynthesis and Applications of Heteropolysaccharides from Lactic Acid Bacteria. Int. Dairy J..

[69] Leo F., Hashida S., Kumagai D., Uchida K., Motoshima H., Arai I., Asakuma S., Fukuda K., Urashima T. (2007). Studies on a Neutral Exopolysaccharide of *Lactobacillus fermentum* TDS030603. J. Appl. Glycosci..

[70] Ruas-Madiedo P., Tuinier R., Kanning M., Zoon P. (2002). Role of Exopolysaccharides Produced by Lactococcus lactis subsp. cremoris on the Viscosity of Fermented Milks. Int. Dairy J..

[71] Ahmed Z., Wang Y., Anjum N., Ahmad H., Ahmad A., Raza M. (2013). Characterization of New Exopolysaccharides Produced by Coculturing of *L. kefiranofaciens* with Yoghurt Strains. Int. J. Biol. Macromol..

[72] Bertsch A., Roy D., LaPointe G. (2019). Enhanced Exopolysaccharide Production by *Lactobacillus rhamnosus* in Co-Culture with *Saccharomyces cerevisiae*. Appl. Sci..

[73] Yu Y.J., Chen Z., Chen P.T., Ng I.S. (2018). Production, Characterization and Antibacterial Activity of Exopolysaccharide from a Newly Isolated Weissella Cibaria under Sucrose Effect. J. Biosci. Bioeng..

[74] Zannini E., Mauch A., Galle S., Gänzle M., Coffey A., Arendt E.K., Taylor J.P., Waters D.M. (2013). Barley Malt Wort Fermentation by Exopolysaccharide-Forming Weissella Cibaria MG1 for the Production of a Novel Beverage. J. Appl. Microbiol..

[75] Dertli E., Mercan E., Arici M., Yilmaz M.T., Sağdiç O. (2016). Characterisation of Lactic Acid Bacteria from Turkish Sourdough and Determination of Their Exopolysaccharide (EPS) Production Characteristics. LWT-Food Sci. Technol..

[76] Imran M.Y.M., Reehana N., Jayaraj K.A., Ahamed A.A.P., Dhanasekaran D., Thajuddin N., Alharbi N.S., Muralitharan G. (2016). Statistical Optimization of Exopolysaccharide Production by Lactobacillus Plantarum NTMI05 and NTMI20. Int. J. Biol. Macromol..

[77] Abdalrahim S., Zohri A.N.A., Khider M., Kamal El-Dean A.M., Abulreesh H.H., Ahmad I., Elbanna K. (2019). Phenotypic and Genotypic Characterization of Exopolysaccharide Producing Bacteria Isolated from Fermented Fruits, Vegetables and Dairy Products. J. Pure Appl. Microbiol..

[78] Salazar N., Prieto A., Leal J.A., Mayo B., Bada-Gancedo J.C., de los Reyes-Gavilán C.G., Ruas-Madiedo P. (2009). Production of Exopolysaccharides by Lactobacillus and Bifidobacterium Strains of Human Origin, and Metabolic Activity of the Producing Bacteria in Milk. J. Dairy Sci..

[79] Ruas-Madiedo P., Gueimonde M., Arigoni F., De Los Reyes-Gavilán C.G., Margolles A. (2009). Bile Affects the Synthesis of Exopolysaccharides by *Bifidobacterium animalis*. Appl. Environ. Microbiol..

[80] Wu Q., Shah N.P. (2018). Comparative MRNA-Seq Analysis Reveals the Improved EPS Production Machinery in Streptococcus Thermophilus ASCC 1275 during Optimized Milk Fermentation. Front. Microbiol..

[81] Stack H.M., Kearney N., Stanton C., Fitzgerald G.F., Ross R.P. (2010). Association of Beta-Glucan Endogenous Production with Increased Stress Tolerance of Intestinal Lactobacilli. Appl. Environ. Microbiol..

[82] Degeest B., Mozzi F., De Vuyst L. (2002). Effect of Medium Composition and Temperature and PH Changes on Exopolysaccharide Yields and Stability during *Streptococcus thermophilus* LY03 Fermentations. Int. J. Food Microbiol..

[83] Yang Z., Li S., Zhang X., Zeng X., Li D., Zhao Y., Zhang J. (2010). Capsular and Slime-Polysaccharide Production by *Lactobacillus rhamnosus* JAAS8 Isolated from Chinese Sauerkraut: Potential Application in Fermented Milk Products. J. Biosci. Bioeng..

[84] Ahmed Z., Wang Y., Anjum N., Ahmad A., Khan S.T. (2013). Characterization of Exopolysaccharide Produced by *Lactobacillus kefiranofaciens* ZW3 Isolated from Tibet Kefir-Part II. Food Hydrocoll..

[85] Saadat Y.R., Khosroushahi A.Y., Gargari B.P. (2019). A Comprehensive Review of Anticancer, Immunomodulatory and Health Beneficial Effects of the Lactic Acid Bacteria Exopolysaccharides. Carbohydr. Polym..

[86] Ortega-Morales B.O., Santiago-García J.L., Chan-Bacab M.J., Moppert X., Miranda-Tello E., Fardeau M.L., Carrero J.C., Bartolo-Pérez P., Valadéz-González A., Guezennec J. (2007). Characterization of Extracellular Polymers Synthesized by Tropical Intertidal Biofilm Bacteria. J. Appl. Microbiol..

[87] Borucki M.K., Peppin J.D., White D., Loge F., Call D.R. (2003). Variation in Biofilm Formation among Strains of *Listeria monocytogenes*. Appl. Environ. Microbiol..

[88] Ma J., Yin R. (2011). Primary Study on Extracellular Polysaccharide Producing Bacteria in Different Environments. J. Anhui Univ. Nat. Sci. Ed..

[89] Lauer Cruz K., de Souza da Motta A. (2019). Characterization of Biofilm Production by *Pseudomonas fluorescens* Isolated from Refrigerated Raw Buffalo Milk. J. Food Sci. Technol..

[90] Ates O. (2015). Systems Biology of Microbial Exopolysaccharides Production. Front. Bioeng. Biotechnol..

[91] Teusink B., Wiersma A., Jacobs L., Notebaart R., Smid E. (2009). Understanding the Adaptive Growth Strategy of *Lactobacillus plantarum* by In Silico Optimisation. PLoS Comput. Biol..

[92] Siezen R.J., van Hylckama Vlieg J.E.T. (2011). Genomic Diversity and Versatility of *Lactobacillus plantarum*, a Natural Metabolic Engineer. Microb. Cell Fact..

[93] Hao P., Zheng H., Yu Y., Ding G., Gu W., Chen S., Yu Z., Ren S., Oda M., Konno T. (2011). Complete Sequencing and Pan-Genomic Analysis of Lactobacillus delbrueckii subsp. bulgaricus Reveal Its Genetic Basis for Industrial Yogurt Production. PLoS ONE.

[94] Jung J.Y., Lee S.H., Kim J.M., Park M.S., Bae J.W., Hahn Y., Madsen E.L., Jeon C.O. (2011). Metagenomic Analysis of Kimchi, a Traditional Korean Fermented Food. Appl. Environ. Microbiol..

[95] Sieuwerts S., Molenaar D., Van Hijum S.A.F.T., Beerthuyzen M., Stevens M.J.A., Janssen P.W.M., Ingham C.J., De Bok F.A.M., De Vos W.M., Van Hylckama Vlieg J.E.T. (2010). Mixed-Culture Transcriptome Analysis Reveals the Molecular Basis of Mixed-Culture Growth in *Streptococcus thermophilus* and *Lactobacillus bulgaricus*. Appl. Environ. Microbiol..

[96] Bouzar F., Cerning J., Desmazeaud M. (1997). Exopolysaccharide Production and Texture-Promoting Abilities of Mixed-Strain Starter Cultures in Yogurt Production. J. Dairy Sci..

[97] Rimada P.S., Abraham A.G. (2003). Comparative Study of Different Methodologies to Determine the Exopolysaccharide Produced by Kefir Grains in Milk and Whey. Lait.

[98] Notararigo S., Nácher-Vázquez M., Ibarburu I., Werning M., De Palencia P.F., Dueñas M.T., Aznar R., López P., Prieto A. (2013). Comparative Analysis of Production and Purification of Homo- and Hetero-Polysaccharides Produced by Lactic Acid Bacteria. Carbohydr. Polym..

[99] Li C., Li W., Chen X., Feng M., Rui X., Jiang M., Dong M. (2014). Microbiological, Physicochemical and Rheological Properties of Fermented Soymilk Produced with Exopolysaccharide (EPS) Producing Lactic Acid Bacteria Strains. LWT-Food Sci. Technol..

[100] Abdhul K., Ganesh M., Shanmughapriya S., Kanagavel M., Anbarasu K., Natarajaseenivasan K. (2014). Antioxidant Activity of Exopolysaccharide from Probiotic Strain *Enterococcus faecium* (BDU7) from Ngari. Int. J. Biol. Macromol..

[101] Donnarumma G., Molinaro A., Cimini D., De Castro C., Valli V., De Gregorio V., De Rosa M., Schiraldi C. (2014). *Lactobacillus crispatus* L1: High Cell Density Cultivation and Exopolysaccharide Structure Characterization to Highlight Potentially Beneficial Effects against Vaginal Pathogens. BMC Microbiol..

[102] Pino A., Bartolo E., Caggia C., Cianci A., Randazzo C.L. (2019). Detection of Vaginal Lactobacilli as Probiotic Candidates. Sci. Rep..

[103] Behare P.V., Singh R., Nagpal R., Rao K.H. (2013). Exopolysaccharides Producing *Lactobacillus fermentum* Strain for Enhancing Rheological and Sensory Attributes of Low-Fat Dahi. J. Food Sci. Technol..

[104] Dimopoulou M., Vuillemin M., Campbell-Sills H., Lucas P.M., Ballestra P., Miot-Sertier C., Favier M., Coulon J., Moine V., Doco T. (2014). Exopolysaccharide (EPS) Synthesis by *Oenococcus oeni*: From Genes to Phenotypes. PLoS ONE.

[105] Ruijssenaars H.J., Stingele F., Hartmans S. (2000). Biodegradability of Food-Associated Extracellular Polysaccharides. Curr. Microbiol..

[106] Katina K., Maina N.H., Juvonen R., Flander L., Johansson L., Virkki L., Tenkanen M., Laitila A. (2009). In Situ Production and Analysis of *Weissella confusa* Dextran in Wheat Sourdough. Food Microbiol..

[107] Yilmaz M.T., Dertli E., Toker O.S., Tatlisu N.B., Sagdic O., Arici M. (2015). Effect of in Situ Exopolysaccharide Production on Physicochemical, Rheological, Sensory, and Microstructural Properties of the Yogurt Drink Ayran: An Optimization Study Based on Fermentation Kinetics. J. Dairy Sci..

[108] Angelin J., Kavitha M. (2020). Exopolysaccharides from Probiotic Bacteria and Their Health Potential. Int. J. Biol. Macromol..

[109] Wang K., Niu M., Song D., Song X., Zhao J., Wu Y., Lu B., Niu G. (2020). Preparation, Partial Characterization and Biological Activity of Exopolysaccharides Produced from *Lactobacillus fermentum* S1. J. Biosci. Bioeng..

[110] Zannini E., Waters D.M., Coffey A., Arendt E.K. (2016). Production, Properties, and Industrial Food Application of Lactic Acid Bacteria-Derived Exopolysaccharides. Appl. Microbiol. Biotechnol..

[111] Sharifi-Rad M., Anil Kumar N.V., Zucca P., Varoni E.M., Dini L., Panzarini E., Rajkovic J., Tsouh Fokou P.V., Azzini E., Peluso I. (2020). Lifestyle, Oxidative Stress, and Antioxidants: Back and Forth in the Pathophysiology of Chronic Diseases. Front. Physiol..

[112] Liu Z., Ren Z., Zhang J., Chuang C.C., Kandaswamy E., Zhou T., Zuo L. (2018). Role of ROS and Nutritional Antioxidants in Human Diseases. Front. Physiol..

[113] Mishra V., Shah C., Mokashe N., Chavan R., Yadav H., Prajapati J. (2015). Probiotics as Potential Antioxidants: A Systematic Review. J. Agric. Food Chem..

[114] Prete R., Garcia-Gonzalez N., Di Mattia C.D., Corsetti A., Battista N. (2020). Food-Borne *Lactiplantibacillus plantarum* Protect Normal Intestinal Cells against Inflammation by Modulating Reactive Oxygen Species and IL-23/IL-17 Axis. Sci. Rep..

[115] Zhang L., Liu C., Li D., Zhao Y., Zhang X., Zeng X., Yang Z., Li S. (2013). Antioxidant Activity of an Exopolysaccharide Isolated from *Lactobacillus plantarum* C88. Int. J. Biol. Macromol..

[116] Seo B.-J., Bajpai V.K., Rather I.A., Park Y.-H. (2015). Partially Purified Exopolysaccharide from *Lactobacillus plantarum* YML009 with Total Phenolic Content, Antioxidant and Free Radical Scavenging Efficacy. Indian J. Pharm. Educ. Res..

[117] Trabelsi I., Ktari N., Ben Slima S., Triki M., Bardaa S., Mnif H., Ben Salah R. (2017). Evaluation of Dermal Wound Healing Activity and in Vitro Antibacterial and Antioxidant Activities of a New Exopolysaccharide Produced by *Lactobacillus* sp.Ca6. Int. J. Biol. Macromol..

[118] Şengül N., Aslím B., Uçar G., Yücel N., Işik S., Bozkurt H., Sakaoğullarí Z., Atalay F. (2006). Effects of Exopolysaccharide-Producing Probiotic Strains on Experimental Colitis in Rats. Dis. Colon Rectum.

[119] Liu C.F., Tseng K.C., Chiang S.S., Lee B.H., Hsu W.H., Pan T.M. (2011). Immunomodulatory and Antioxidant Potential of Lactobacillus Exopolysaccharides. J. Sci. Food Agric..

[120] Polak-Berecka M., Waśko A., Szwajgier D., Choma A. (2013). Bifidogenic and Antioxidant Activity of Exopolysaccharides Produced by *Lactobacillus rhamnosus* E/N Cultivated on Different Carbon Sources. Polish J. Microbiol..

[121] Li J.Y., Jin M.M., Meng J., Gao S.M., Lu R.R. (2013). Exopolysaccharide from *Lactobacillus planterum* LP6: Antioxidation and the Effect on Oxidative Stress. Carbohydr. Polym..

[122] Li W., Ji J., Chen X., Jiang M., Rui X., Dong M. (2014). Structural Elucidation and Antioxidant Activities of Exopolysaccharides from *Lactobacillus helveticus* MB2-1. Carbohydr. Polym..

[123] Rani R.P., Anandharaj M., David Ravindran A. (2018). Characterization of a Novel Exopolysaccharide Produced by *Lactobacillus gasseri* FR4 and Demonstration of Its in Vitro Biological Properties. Int. J. Biol. Macromol..

[124] Guo Y., Pan D., Li H., Sun Y., Zeng X., Yan B. (2013). Antioxidant and Immunomodulatory Activity of Selenium Exopolysaccharide Produced by *Lactococcus lactis* subsp. lactis. Food Chem..

[125] Dilna S.V., Surya H., Aswathy R.G., Varsha K.K., Sakthikumar D.N., Pandey A., Nampoothiri K.M. (2015). Characterization of an Exopolysaccharide with Potential Health-Benefit Properties from a Probiotic *Lactobacillus plantarum* RJF4. LWT-Food Sci. Technol..

[126] Yılmaz T., Şimşek Ö. (2020). Potential Health Benefits of Ropy Exopolysaccharides Produced by *Lactobacillus plantarum*. Molecules.

[127] Jiang Y., Jiang X., Wang P., Mou H., Hu X., Liu S. (2008). The Antitumor and Antioxidative Activities of Polysaccharides Isolated from *Isaria farinosa* B05. Microbiol. Res..

[128] Pan D., Mei X. (2010). Antioxidant Activity of an Exopolysaccharide Purified from *Lactococcus lactis* subsp. lactis 12. Carbohydr. Polym..

[129] Zhang J., Zhao X., Jiang Y., Zhao W., Guo T., Cao Y., Teng J., Hao X., Zhao J., Yang Z. (2017). Antioxidant Status and Gut Microbiota Change in an Aging Mouse Model as Influenced by Exopolysaccharide Produced by *Lactobacillus plantarum* YW11 Isolated from Tibetan Kefir. J. Dairy Sci..

[130] Li B., Du P., Smith E.E., Wang S., Jiao Y., Guo L., Huo G., Liu F. (2019). In Vitro and in Vivo Evaluation of an Exopolysaccharide Produced by *Lactobacillus helveticus* KLDS1.8701 for the Alleviative Effect on Oxidative Stress. Food Funct..

[131] Tok E., Aslim B. (2010). Cholesterol Removal by Some Lactic Acid Bacteria That Can Be Used as Probiotic. Microbiol. Immunol..

[132] Costabile A., Buttarazzi I., Kolida S., Quercia S., Baldini J., Swann J.R., Brigidi P., Gibson G.R. (2017). An in Vivo Assessment of the Cholesterol-Lowering Efficacy of *Lactobacillus plantarum* ECGC 13110402 in Normal to Mildly Hypercholesterolaemic Adults. PLoS ONE.

[133] Bhat B., Bajaj B.K. (2019). Hypocholesterolemic Potential and Bioactivity Spectrum of an Exopolysaccharide from a Probiotic Isolate *Lactobacillus paracasei* M7. Bioact. Carbohydr. Diet. Fibre.

[134] Nakajima H., Suzuki Y., Hirota T. (1992). Cholesterol Lowering Activity of Ropy Fermented Milk. J. Food Sci..

[135] Ai L., Zhang H., Guo B., Chen W., Wu Z., Wu Y. (2008). Preparation, Partial Characterization and Bioactivity of Exopolysaccharides from *Lactobacillus casei* LC2W. Carbohydr. Polym..

[136] Lim J., Kale M., Kim D.H., Kim H.S., Chon J.W., Seo K.H., Lee H.G., Yokoyama W., Kim H. (2017). Antiobesity Effect of Exopolysaccharides Isolated from Kefir Grains. J. Agric. Food Chem..

[137] Wang Y., Xu N., Xi A., Ahmed Z., Zhang B., Bai X. (2009). Effects of *Lactobacillus plantarum* MA2 Isolated from Tibet Kefir on Lipid Metabolism and Intestinal Microflora of Rats Fed on High-Cholesterol Diet. Appl. Microbiol. Biotechnol..

[138] Akalin A.S., Gönç S., Düzel S. (1997). Influence of Yogurt and Acidophilus Yogurt on Serum Cholesterol Levels in Mice. J. Dairy Sci..

[139] Liu J.-R., Wang S.-Y., Chen M.-J., Chen H.-L., Yueh P.-Y., Lin C.-W. (2006). Hypocholesterolaemic Effects of Milk-Kefir and Soyamilk-Kefir in Cholesterol-Fed Hamsters. Br. J. Nutr..

[140] Gunness P., Gidley M.J. (2010). Mechanisms Underlying the Cholesterol-Lowering Properties of Soluble Dietary Fibre Polysaccharides. Food Funct..

[141] Lye H.S., Rusul G., Liong M.T. (2010). Removal of Cholesterol by Lactobacilli via Incorporation and Conversion to Coprostanol. J. Dairy Sci..

[142] Prete R., Long S.L., Gallardo A.L., Gahan C.G., Corsetti A., Joyce S.A. (2020). Beneficial Bile Acid Metabolism from *Lactobacillus plantarum* of Food Origin. Sci. Rep..

[143] Jeong D., Kim D.H., Kang I.B., Kim H., Song K.Y., Kim H.S., Seo K.H. (2017). Characterization and Antibacterial Activity of a Novel Exopolysaccharide Produced by *Lactobacillus kefiranofaciens* DN1 Isolated from Kefir. Food Control.

[144] Ayyash M., Abu-Jdayil B., Itsaranuwat P., Galiwango E., Tamiello-Rosa C., Abdullah H., Esposito G., Hunashal Y., Obaid R.S., Hamed F. (2020). Characterization, Bioactivities, and Rheological Properties of Exopolysaccharide Produced by Novel Probiotic *Lactobacillus plantarum* C70 Isolated from Camel Milk. Int. J. Biol. Macromol..

[145] Dertli E., Colquhoun I.J., Gunning A.P., Bongaerts R.J., Le Gall G., Bonev B.B., Mayer M.J., Narbad A. (2013). Structure and Biosynthesis of Two Exopolysaccharides Produced by *Lactobacillus johnsonii* FI9785. J. Biol. Chem..

[146] Spanò A., Laganà P., Visalli G., Maugeri T.L., Gugliandolo C. (2016). In Vitro Antibiofilm Activity of an Exopolysaccharide from the Marine Thermophilic *Bacillus licheniformis* T14. Curr. Microbiol..

[147] Xing K., Chen X.G., Kong M., Liu C.S., Cha D.S., Park H.J. (2009). Effect of Oleoyl-Chitosan Nanoparticles as a Novel Antibacterial Dispersion System on Viability, Membrane Permeability and Cell Morphology of Escherichia Coli and *Staphylococcus aureus*. Carbohydr. Polym..

[148] Rajoka M.S.R., Jin M., Haobin Z., Li Q., Shao D., Jiang C., Huang Q., Yang H., Shi J., Hussain N. (2018). Functional Characterization and Biotechnological Potential of Exopolysaccharide Produced by *Lactobacillus rhamnosus* Strains Isolated from Human Breast Milk. LWT-Food Sci. Technol..

[149] Sarikaya H., Aslim B., Yuksekdag Z. (2017). Assessment of Anti-Biofilm Activity and Bifidogenic Growth Stimulator (BGS) Effect of Lyophilized Exopolysaccharides (l-EPSs) from Lactobacilli Strains. Int. J. Food Prop..

[150] Abid Y., Casillo A., Gharsallah H., Joulak I., Lanzetta R., Corsaro M.M., Attia H., Azabou S. (2018). Production and Structural Characterization of Exopolysaccharides from Newly Isolated Probiotic Lactic Acid Bacteria. Int. J. Biol. Macromol..

[151] Rosca I., Petrovici A.R., Peptanariu D., Nicolescu A., Dodi G., Avadanei M., Ivanov I.C., Bostanaru A.C., Mares M., Ciolacu D. (2018). Biosynthesis of Dextran by *Weissella confusa* and Its In Vitro Functional Characteristics. Int. J. Biol. Macromol..

[152] Kanmani P., Suganya K., Satish Kumar R., Yuvaraj N., Pattukumar V., Paari K.A., Arul V. (2013). Synthesis and Functional Characterization of Antibiofilm Exopolysaccharide Produced by *Enterococcus faecium* Mc13 Isolated from the Gut of Fish. Appl. Biochem. Biotechnol..

[153] Jiang P., Li J., Han F., Duan G., Lu X., Gu Y., Yu W. (2011). Antibiofilm Activity of an Exopolysaccharide from Marine Bacterium *Vibrio* sp. QY101. PLoS ONE.

[154] Garcia-Castillo V., Marcial G., Albarracín L., Tomokiyo M., Clua P., Takahashi H., Kitazawa H., Garcia-Cancino A., Villena J. (2020). The Exopolysaccharide of *Lactobacillus fermentum* UCO-979C Is Partially Involved in Its Immunomodulatory Effect and Its Ability to Improve the Resistance against Helicobacter Pylori Infection. Microorganisms.

[155] Korcz E., Kerényi Z., Varga L. (2018). Dietary Fibers, Prebiotics, and Exopolysaccharides Produced by Lactic Acid Bacteria: Potential Health Benefits with Special Regard to Cholesterol-Lowering Effects. Food Funct..

[156] Gagliardi A., Totino V., Cacciotti F., Iebba V., Neroni B., Bonfiglio G., Trancassini M., Passariello C., Pantanella F., Schippa S. (2018). Rebuilding the Gut Microbiota Ecosystem. Int. J. Environ. Res. Public Health.

[157] Gibson G.R., Roberfroid M.B. (1995). Dietary Modulation of the Human Colonic Microbiota: Introducing the Concept of Prebiotics. J. Nutr..

[158] Gibson G.R., Hutkins R., Sanders M.E., Prescott S.L., Reimer R.A., Salminen S.J., Scott K., Stanton C., Swanson K.S., Cani P.D. (2017). Expert Consensus Document: The International Scientific Association for Probiotics and Prebiotics (ISAPP) Consensus Statement on the Definition and Scope of Prebiotics. Nat. Rev. Gastroenterol. Hepatol..

[159] Salazar N., Gueimonde M., de los Reyes-Gavilán C.G., Ruas-Madiedo P. (2016). Exopolysaccharides Produced by Lactic Acid Bacteria and Bifidobacteria as Fermentable Substrates by the Intestinal Microbiota. Crit. Rev. Food Sci. Nutr..

[160] Dal Bello F., Walter J., Hertel C., Hammes W.P. (2001). In Vitro Study of Prebiotic Properties of Levan-Type Exopolysaccharides from Lactobacilli and Non-Digestible Carbohydrates Using Denaturing Gradient Gel Electrophoresis. Syst. Appl. Microbiol..

[161] Hongpattarakere T., Cherntong N., Wichienchot S., Kolida S., Rastall R.A. (2012). In Vitro Prebiotic Evaluation of Exopolysaccharides Produced by Marine Isolated Lactic Acid Bacteria. Carbohydr. Polym..

[162] Das D., Baruah R., Goyal A. (2014). A Food Additive with Prebiotic Properties of an α-d-Glucan from *Lactobacillus plantarum* DM5. Int. J. Biol. Macromol..

[163] Tang W., Zhou J., Xu Q., Dong M., Fan X., Rui X., Zhang Q., Chen X., Jiang M., Wu J. (2020). In Vitro Digestion and Fermentation of Released Exopolysaccharides (r-EPS) from Lactobacillus delbrueckii ssp. bulgaricus SRFM-1. Carbohydr. Polym..

[164] Tang W., Han S., Zhou J., Xu Q., Dong M., Fan X., Rui X., Zhang Q., Chen X., Jiang M. (2020). Selective Fermentation of *Lactobacillus delbrueckii* ssp. bulgaricus SRFM-1 Derived Exopolysaccharide by Lactobacillus and Streptococcus Strains Revealed Prebiotic Properties. J. Funct. Foods.

[165] Russo P., López P., Capozzi V., de Palencia P.F., Dueñas M.T., Spano G., Fiocco D. (2012). Beta-Glucans Improve Growth, Viability and Colonization of Probiotic Microorganisms. Int. J. Mol. Sci..

[166] Lindström C., Holst O., Nilsson L., Öste R., Andersson K.E. (2012). Effects of *Pediococcus parvulus* 2.6 and Its Exopolysaccharide on Plasma Cholesterol Levels and Inflammatory Markers in Mice. AMB Express.

[167] Lambo-Fodje A.M., Öste R., Nyman M.E.G.-L. (2006). Short-Chain Fatty Acid Formation in the Hindgut of Rats Fed Native and Fermented Oat Fibre Concentrates. Br. J. Nutr..

[168] Fraher M.H., O’Toole P.W., Quigley E.M.M. (2012). Techniques Used to Characterize the Gut Microbiota: A Guide for the Clinician. Nat. Rev. Gastroenterol. Hepatol..

[169] Hyötyläinen T. (2012). Novel Methodologies in Metabolic Profiling with a Focus on Molecular Diagnostic Applications. Expert Rev. Mol. Diagn..

[170] Xu Y., Cui Y., Yue F., Liu L., Shan Y., Liu B., Zhou Y., Lü X. (2019). Exopolysaccharides Produced by Lactic Acid Bacteria and Bifidobacteria: Structures, Physiochemical Functions and Applications in the Food Industry. Food Hydrocoll..

[171] Di Cagno R., De Pasquale I., De Angelis M., Buchin S., Rizzello C.G., Gobbetti M. (2014). Use of Microparticulated Whey Protein Concentrate, Exopolysaccharide-Producing *Streptococcus thermophilus*, and Adjunct Cultures for Making Low-Fat Italian Caciotta-Type Cheese. J. Dairy Sci..

[172] Purohit D.H., Hassan A.N., Bhatia E., Zhang X., Dwivedi C. (2009). Rheological, Sensorial, and Chemopreventive Properties of Milk Fermented with Exopolysaccharide-Producing Lactic Cultures. J. Dairy Sci..

[173] Amatayakul T., Halmos A.L., Sherkat F., Shah N.P. (2006). Physical Characteristics of Yoghurts Made Using Exopolysaccharide-Producing Starter Cultures and Varying Casein to Whey Protein Ratios. Int. Dairy J..

[174] Han X., Yang Z., Jing X., Yu P., Zhang Y., Yi H., Zhang L. (2016). Improvement of the Texture of Yogurt by Use of Exopolysaccharide Producing Lactic Acid Bacteria. BioMed Res. Int..

[175] Petry S., Furlan S., Waghorne E., Saulnier L., Cerning J., Maguin E. (2003). Comparison of the Thickening Properties of Four Lactobacillus delbrueckii subsp. bulgaricus Strains and Physicochemical Characterization of Their Exopolysaccharides. FEMS Microbiol. Lett..

[176] Ayyash M., Abu-Jdayil B., Hamed F., Shaker R. (2018). Rheological, Textural, Microstructural and Sensory Impact of Exopolysaccharide-Producing *Lactobacillus plantarum* Isolated from Camel Milk on Low-Fat Akawi Cheese. LW-Food Sci. Technol..

[177] Ale E.C., Perezlindo M.J., Pavón Y., Peralta G.H., Costa S., Sabbag N., Bergamini C., Reinheimer J.A., Binetti A.G. (2016). Technological, Rheological and Sensory Characterizations of a Yogurt Containing an Exopolysaccharide Extract from *Lactobacillus fermentum* Lf2, a New Food Additive. Food Res. Int..

[178] London L.E.E., Chaurin V., Auty M.A.E., Fenelon M.A., Fitzgerald G.F., Ross R.P., Stanton C. (2015). Use of *Lactobacillus mucosae* DPC 6426, an Exopolysaccharide-Producing Strain, Positively Influences the Techno-Functional Properties of Yoghurt. Int. Dairy J..

[179] Prasanna P.H.P., Grandison A.S., Charalampopoulos D. (2013). Microbiological, Chemical and Rheological Properties of Low Fat Set Yoghurt Produced with Exopolysaccharide (EPS) Producing *Bifidobacterium* Strains. Food Res. Int..

[180] Medrano M., Hamet M.F., Abraham A.G., Pérez P.F. (2009). Kefiran Protects Caco-2 Cells from Cytopathic Effects Induced by *Bacillus cereus* Infection. Antonie van Leeuwenhoek Int. J. Gen. Mol. Microbiol..

[181] Escalante A., Elena Rodríguez M., Martínez A., López-Munguía A., Bolívar F., Gosset G. (2004). Characterization of Bacterial Diversity in Pulque, a Traditional Mexican Alcoholic Fermented Beverage, as Determined by 16S RDNA Analysis. FEMS Microbiol. Lett..

[182] Behare P., Singh R., Singh R.P. (2009). Exopolysaccharide-Producing Mesophilic Lactic Cultures for Preparation of Fat-Free Dahi-an Indian Fermented Milk. J. Dairy Res..

[183] Hassan A.N. (2008). ADSA Foundation Scholar Award: Possibilities and Challenges of Exopolysaccharide-Producing Lactic Cultures in Dairy Foods. J. Dairy Sci..

[184] Costa N.E., Hannon J.A., Guinee T.P., Auty M.A.E., McSweeney P.L.H., Beresford T.P. (2010). Effect of Exopolysaccharide Produced by Isogenic Strains of *Lactococcus lactis* on Half-Fat Cheddar Cheese. J. Dairy Sci..

[185] Hassan A.N., Corredig M., Frank J.F., Elsoda M. (2004). Microstructure and Rheology of an Acid-Coagulated Cheese (Karish) Made with an Exopolysaccharide-Producing *Streptococcus thermophilus* Strain and Its Exopolysaccharide Non-Producing Genetic Variant. J. Dairy Res..

[186] Characklis W.G., Wilderer P.A. (1989). The Structure and Function of Biofilms.

